# Context-Dependent Spatial and Functional Roles of CD44 Across Pancreatic Ductal Adenocarcinoma States

**DOI:** 10.3390/cancers18203410

**Published:** 2026-10-21

**Authors:** Bin Fu, Yingying Tan, Yaxin Chen, Yongren Su, Mengran Cao, Dashan Tan, Meng Zhang, Xuewei Chen, Rong Hu

**Affiliations:** 1 State Key Laboratory of Natural Medicines, School of Basic Medicine and Clinical Pharmacy, China Pharmaceutical University, Nanjing 211198, China; fubin7369@gmail.com (B.F.); tamyying@163.com (Y.T.); cyxcpu@163.com (Y.C.); cameraaaa@126.com (M.C.); xiangyuzeds@163.com (D.T.); m_zhang2001@163.com (M.Z.); 18255020274@163.com (X.C.); 2 State Key Laboratory of Medicinal Chemical Biology and College of Pharmacy, Nankai University, Tianjin 300071, China; syr19902181026@163.com

**Keywords:** Pancreatic Ductal Adenocarcinoma (PDAC), CD44, cellular plasticity, spatial organization, hyaluronan

## Abstract

**Background/Objectives**: Pancreatic ductal adenocarcinoma (PDAC) comprises distinct cellular states characterized by different differentiation programs and therapeutic responses. How a cell-surface receptor adapts its function across these states remains poorly understood. Here, we investigated whether the spatial organization and functional output of CD44 vary across PDAC cellular states. **Methods**: CD44 expression and spatial distribution were examined in human PDAC tissues and PDAC cell models representing different differentiation states. The functional roles of CD44 were investigated using genetic depletion, transcriptomic analysis, migration and invasion assays, and xenograft and orthotopic models. Hyaluronan (HA) responsiveness and downstream signaling were assessed following exogenous HA stimulation, and the therapeutic effects of HA synthesis inhibition with 4-methylumbelliferone (4-MU) were evaluated alone or in combination with gemcitabine. **Results**: In differentiated PDAC tissues and classical PDAC models, CD44 was predominantly localized to the basolateral membrane and spatially segregated from E-cadherin at cell–cell junctions, consistent with a role in epithelial organization and cell–matrix interactions. In contrast, poorly differentiated PDAC tissues and basal-like models exhibited increased CD44 abundance, E-cadherin loss, and redistribution of CD44 along the cell periphery. Genetic depletion of *CD44* preferentially impaired malignant phenotypes in basal-like models. Mechanistically, basal-like cells exhibited enhanced CD44-dependent responsiveness to extracellular HA, accompanied by activation of EMT-associated signaling and destabilization of E-cadherin. Inhibition of HA synthesis with 4-MU preferentially suppressed basal-like PDAC models and enhanced gemcitabine sensitivity, with these effects further supported in orthotopic models. **Conclusions**: These findings suggest that the functional output of CD44 in PDAC is influenced not only by its expression level but also by cellular context and spatial organization. The context-dependent responsiveness of the HA–CD44 axis may contribute to functional heterogeneity among PDAC states and represents a potential therapeutic vulnerability of basal-like PDAC.

## Simple Summary

Pancreatic ductal adenocarcinoma (PDAC) is a difficult-to-treat cancer in which tumor cells can exist in different states. CD44 is a protein on the surface of cancer cells that helps them interact with their surroundings. However, it is not clear whether CD44 plays the same role in different PDAC cell types. We found that CD44 exhibits distinct spatial organization in differentiated versus poorly differentiated PDAC tissues and cells. In differentiated PDAC, CD44 shows an anchoring-associated localization at the cell–matrix interface and is associated with epithelial organization and basement membrane attachment. In contrast, poorly differentiated and basal-like PDAC cells show increased and redistributed CD44, concomitant loss of E-cadherin, and function as sensors for extracellular hyaluronan (HA). Genetic depletion of *CD44* reduced the aggressive traits of these basal-like cells. Moreover, blocking HA production preferentially slowed the growth of basal-like PDAC cells and enhanced their sensitivity to the chemotherapy drug gemcitabine. These findings suggest that CD44’s role depends on the cellular state and that the HA–CD44 pathway may represent a promising therapeutic strategy for more aggressive PDAC subtypes.

## 1. Introduction

Pancreatic ductal adenocarcinoma (PDAC) is a highly aggressive malignancy with a five-year survival rate of 13% [1]. Beyond its poor clinical outcome, PDAC is characterized by marked cellular heterogeneity and phenotypic plasticity, which contribute to disease progression and therapeutic resistance. Traditionally, pancreatic ductal adenocarcinoma has been classified histopathologically according to tumor differentiation and glandular architecture, ranging from well-differentiated tumors with relatively preserved gland formation to poorly differentiated tumors characterized by disorganized architecture and loss of epithelial features. These pathological differences are closely associated with tumor aggressiveness and clinical behavior, highlighting the existence of distinct cellular states within PDAC [2,3]. With the development of transcriptomic profiling, this phenotypic heterogeneity has been further characterized at the molecular level. Transcriptomic studies have broadly defined classical and basal-like states, with the former generally exhibiting more differentiated epithelial features and the latter associated with poor differentiation, epithelial–mesenchymal transition (EMT), and aggressive behavior [4,5,6]. These biological differences have important therapeutic implications. The COMPASS trial demonstrated that basal-like PDAC responds more favorably to gemcitabine/nab-paclitaxel, whereas classical tumors derive greater benefit from FOLFIRINOX [7]. Accordingly, considerable efforts have focused on identifying molecular features and signature genes that distinguish these states, including regulators such as TP63, SEMA3A, and SPDEF [8,9,10]. Meanwhile, increasing evidence from single-cell and spatial transcriptomic studies indicates that these states are not rigid entities but exist along a dynamic phenotypic continuum [11,12]. This raises an important question: rather than simply identifying molecules that distinguish tumor states, can a molecule itself acquire distinct functions as tumor cells transition between states? Thus, whether a molecule is expressed at high or low levels may not fully predict its biological function, particularly when its spatial organization and cellular context change. However, whether such context-dependent functional plasticity occurs for individual cell-surface receptors in PDAC remains poorly understood.

CD44 is a cell-surface receptor and the principal receptor for hyaluronan (HA), with established roles in cell adhesion, migration, tumor initiation, metastasis, and therapeutic resistance [13,14,15,16]. CD44 is also widely used as a marker of cancer stem-like populations, and high CD44 expression is frequently associated with mesenchymal or basal-like phenotypes in several cancers, including PDAC [17,18,19,20]. However, most studies have focused on the abundance of CD44 or its association with aggressive tumor phenotypes, while its spatial organization and functional properties across distinct PDAC states remain largely unexplored. Importantly, CD44 is not a universal oncogenic driver; its biological effects can vary across tumor types and cellular contexts [21,22]. These observations raise an important question: can the same cell-surface receptor adopt distinct spatial organizations and functional roles as PDAC cells transition between different phenotypic states? We hypothesized that CD44 may undergo context-dependent functional changes during PDAC differentiation and plasticity.

To address this question, we investigated the spatial distribution and functional consequences of CD44 across PDAC states by integrating clinical specimens, transcriptomic datasets, and complementary genetic, molecular, cellular, and in vivo approaches. We found that CD44 exhibited distinct spatial organization and functional properties between PDAC states. In differentiated, classical cells, CD44 was predominantly localized to the basolateral membrane and spatially segregated from E-cadherin at cell–cell junctions, whereas basal-like cells exhibited E-cadherin loss accompanied by increased and redistributed CD44. *CD44* depletion preferentially impaired malignant phenotypes in basal-like models, in which CD44 also showed enhanced responsiveness to extracellular HA and activation of EMT signaling. Pharmacological inhibition of HA synthesis preferentially suppressed basal-like PDAC and enhanced gemcitabine sensitivity in vitro and in vivo. Together, these findings reveal a state-dependent functional transition of CD44 in PDAC, from a basolateral epithelial-organizing molecule in differentiated tumors to an HA-responsive signaling receptor in poor differentiated tumors. This spatial rewiring of CD44 provides a potential explanation for its divergent functions across PDAC states and identifies the HA–CD44 axis as a state-selective therapeutic vulnerability.

## 2. Materials and Methods

### 2.1. Cell Lines and Culture

Human pancreatic cancer cell lines PANC-1, MIA PaCa-2, and CFPAC-1 were obtained from the Cell Bank of the Shanghai Institute of Biochemistry and Cell Biology, Chinese Academy of Sciences (Shanghai, China). BxPC-3 cells were kindly provided by Prof. Qinglong Guo (China Pharmaceutical University, Nanjing, China), PaSCs cells were kindly provided by Prof. Wei Chen (Tongde hospital of Zhejiang Province, Hangzhou, China) and the mouse PDAC cell line KPC was kindly provided by Prof. Feng Qian (Tsinghua University, Beijing, China). The KPC line was established from tumors of KPC (Kras^LSL-G12D/+; Trp53^LSL-R172H/+; Pdx1-Cre) mice.

PANC-1, MIA PaCa-2, PaSCs and Pan02 cells were maintained in DMEM (Biochannel, Nanjing, China) supplemented with 10% Fetal Bovine Serum (FBS, Royacel, Lanzhou, China) and 1% penicillin–streptomycin (Pen-Strep, Biochannel, Nanjing, China). CFPAC-1 and BxPC-3 cells were cultured in IMDM (KeyGEN BioTECH, Nanjing, China) or RPMI-1640 (Biochannel, Nanjing, China), respectively, with 10% FBS and 1% Pen-Strep. The m231121 cells were maintained in DMEM with 10% FBS and 1% Pen-Strep. All cell lines were cultured at 37 °C in a humidified atmosphere containing 5% CO_2_.

### 2.2. Animal Experiments

Seven-week-old C57BL/6 mice and BALB/c nude male mice were purchased from Hangzhou Ziyuan Experimental Animal Co. Ltd. (Hangzhou, China). All mice were housed under specific pathogen-free conditions and allowed to acclimatize for one week prior to experimentation. Each individual mouse was considered an experimental unit for all statistical analyses. Sample size was determined based on previous studies using similar xenograft models and our preliminary experiments. Mice were randomly assigned to groups using a random number table. All animal experiments were approved by the Animal Welfare and Ethics Committee of China Pharmaceutical University (Approval No. 2024-11-106).

### 2.3. Subcutaneous Xenograft Models

For subcutaneous xenografts, BALB/c nude mice were injected in the axillary region with 5 × 10^6^ MIA PaCa-2 control or *CD44*-knockout cells suspended in PBS. Tumor growth was monitored regularly, and mice were euthanized 28 days post-implantation. Excised tumors were photographed and weighed. Mice were randomly assigned to two groups (n = 8 per group), with no exclusions from analysis.

### 2.4. Orthotopic Pancreatic Tumor Models

Orthotopic pancreatic tumor model was performed as described [23]. C57BL/6 mice were used for orthotopic implantation of Pan02 cells. After one week of acclimatization, mice were anesthetized with isoflurane, and a small incision exposed the spleen and pancreas. Pan02 control or *CD44*-sh cells (1 × 10^7^ cells/mL; 50 µL per mouse) were injected into the pancreatic parenchyma, with successful delivery confirmed by visible pancreatic swelling without leakage. Organs were returned to the abdominal cavity, and incisions were sutured. Mice were monitored and euthanized upon significant weight loss or morbidity. Tumors were collected, photographed, and weighed. Mice were allocated into two groups (control n = 6, *CD44*-sh n = 9), with three deaths in the control group prior to endpoint; all surviving animals were included in analysis.

### 2.5. Lung Metastasis Models

Orthotopic pancreatic tumor model was performed as described [24]. Pan02 or *CD44*-sh cells (1 × 10^6^ cells/mouse) were injected via the tail vein of C57BL/6 mice. Mice were euthanized 50 days later, and lungs were harvested to assess metastatic burden. Mice were randomly assigned into two groups (n = 6 per group), with all included in analysis.

### 2.6. In Vivo Drug Treatment Studies

Orthotopic pancreatic tumor models were established using Pan02, KPC (both in C57BL/6 mice), and MIA PaCa-2 (BALB/c nude mice) as described above. Mice were randomized into four treatment groups: control, gemcitabine (GEM, 10 mg/kg), 4-methylumbelliferone (4-MU, 100 mg/kg), and combination therapy. Treatment started seven days post-implantation and was administered via oral gavage every three days for 21 days. Mice were then euthanized, and tumors and major organs were collected, weighed, and processed. Each group included six mice, with no exclusions from analysis.

### 2.7. Lentiviral-Mediated CD44 Knockdown and Knockout

Small guide RNA (sgRNA) Plasmid targeting human *CD44* (P62224) and short hairpin RNA (shRNA) targeting mouse *CD44* (P74606, P74944) was purchased from MiaoLing Plasmid Sharing Platform (MiaoLingbio, Wuhan, China). Lentiviral particles were produced in HEK293T cells using the packaging plasmids PsPAX2 and pMD2G. Target cells were transduced with the lentivirus and selected with puromycin (1 µg/mL) to establish stable knockdown lines.

### 2.8. Lentiviral-Mediated CD44 Overexpression

The Overexpression Plasmid targeting human (P40982) or mouse *CD44* (P45746) were purchased from MiaoLing Plasmid Sharing Platform. Target cells were transduced with plasmid and Lipo8000 (Beyotime, Shanghai, China) and selected with G418 or puromycin to establish stable overexpression lines.

### 2.9. qRT-PCR

Experiment was performed as described previously [25]. Total RNA was extracted using TRIzol reagent (catalog no. 15596026, Life technologies, Waltham, MA, USA) as described [26]. cDNA synthesis was performed by EasyScript First-Strand cDNA Synthesis Super Mix (catalog no. AE301, Transgen, Beijing, China) with a reverse PCR instrument (TP600, TAKARA, Shiga, Japan). Quantitative PCR was performed using Power SYBR Green PCR Master Mix (catalog no. Q421-02, Vazyme, Nanjing, China) on a CFX96 Real-Time PCR System (MA-6000, Suzhou Molarray Co., Ltd. Suzhou, China). Primer sequences are listed in Supplementary Table S1.

### 2.10. Immunoblot Analysis

Immunoblotting was performed as described previously [26]. Briefly, human and mouse cell lysates were obtained by using RIPA lysis buffer following the manufacturer’s instructions (P0013B, Beyotime, Shanghai, China), and protein concentrations were determined by a BCA protein assay kit (P0012S, Beyotime, Shanghai, China). Lysates (30 mg of total protein), as well as lysates (10 µL) were then separated by SDS-PAGE gel [separating gel: 1.9 mL ddH_2_O, 1.7 mL Acryl/Bis 30% Solution, 1.3 mL 1.5 mol/L Tris-HCl (pH 8.8), 0.05 mL 10% SDS (*w*/*v*), 0.05 mL 10% APS substitute (*w*/*v*), and 0.002 mL TEMED; spacer gel: 1.4 mL ddH_2_O, 0.33 mL Acryl/Bis 30% Solution, 0.25 mL 1.0 mol/L Tris-HCl (pH 6.8), 0.02 mL 10% SDS (*w*/*v*), 0.02 mL 10% APS substitute (*w*/*v*), and 0.002 mL TEMED] and transferred to polyvinylidene difluoride membrane for immunoblotting analysis using the following antibodies: anti-CD44 (CST, 37259S, Danvers, MA, USA), anti-E-cadherin (HUABIO, HA723564, Hangzhou, China), anti-N-cadherin (HUABIO, ET1607-37, Hangzhou, China), anti-ZEB1 (ImmunoWay, YM8138, San Jose, CA, USA), anti-VIMENTIN (HUABIO, ET1610-39, Hangzhou, China), anti-SNAI1 (ImmunoWay, YM8754, San Jose, CA, USA), anti-β-catenin (Abways, CY3523, Shanghai, China), anti-Src (ImmunoWay, YT1136, San Jose, CA, USA), anti-p-Src (ImmunoWay, YM8417, San Jose, CA, USA), anti-ERK (HUABIO, ET1601-29, Hangzhou, China), anti-p-ERK (HUABIO, ET1610-13, Hangzhou, China),anti-AKT (Proteintech, 60203-2-Ig, Wuhan, China), anti-FAK (Abways, CY5464, Shanghai, China), anti-TGFβR (Proteintech, 30117-1-AP, Wuhan, China), anti-COLA1 (Proteintech, 14695-1-AP, Wuhan, China) and anti-Hakai (ImmunoWay, YN6243, San Jose, CA, USA), with anti-GAPDH (ImmunoWay, YM8394, San Jose, CA, USA) used as loading control. All antibodies were diluted at a ratio of 1:1000 and relative protein expression levels were quantified using ImageJ software (version 1.52a). The intensity of each target protein band was normalized to the corresponding loading control. For phosphorylated proteins, the phosphorylated protein level was normalized to the corresponding total protein level, where applicable. Quantification was based on 3 independent experiments, as indicated in the figure legends. Protein samples requiring membrane fractionation were processed according to the membrane fractionation kit protocol (Beyotime, P0033, Shanghai, China). For Src kinase activating experiment, PP2 (GlpBio, GC10344, Montclair, CA, USA)-a Src kinase inhibitor was pretreated or not 30 min before adding hyaluronic acid (50 µg/mL) (GlpBio, GC32732, Montclair, CA, USA). Extract the protein follow the standard lysis protocols at 0, 2, 5, 10, 15 and 30 min after adding hyaluronic acid. For the PaSCs condition culture experiment, after 48 h of culture, the supernatant from PaSCs cells was centrifuged and filtered, then added to MIA PaCa-2 and CFPAC-1 cells. Protein samples were collected after stimulation for 0, 2, 5, 10, and 15 min. For MIA PaCa-2 cells, additional samples were collected after 1, 2, and 4 h of stimulation. The original blot can be found in Supplementary Files.

### 2.11. Co-Immunoprecipitation (Co-IP)

Cells were lysed in RIPA buffer on ice for 30 min. In 293T cells, the following groups were used: control, PP2 (10 µM), HA (50 µg/mL), and HA (50 µg/mL) + hyaluronidase (10 µg/ml) (Psaitong, H10960, Beijing, China). In MIA PaCa-2 cells, the groups included control, HA (50 µg/mL), and MIA PaCa-2-*CD44*-KO cells. In CFPAC-1 cells, control and HA (50 µg/mL) were used. The lysates were cleared by centrifugation at 12,000× *g* for 15 min at 4 °C. Co-immunoprecipitation was performed using Protein A/G Magnetic Beads (MedChemExpress, Monmouth Junction, NJ, USA) according to the manufacturer’s protocol. Immunoprecipitated proteins were eluted with SDS-PAGE sample buffer, boiled, and analyzed by Western blotting. Protein interactions were detected using anti-E-cadherin (HUABIO, HA723564, Hangzhou, China) and anti-Ubiquitin (Bioworld, BZ16631, Minneapolis, MN, USA) antibodies, followed by chemiluminescence detection.

### 2.12. ELISA

Cell culture supernatants from MIA PaCa-2 and CFPAC-1 cells were collected at 5 min, 15 min, 60 min, 6 h, 12 h, and 48 h after the indicated treatments and clarified by centrifugation at 1000× *g* for 15 min at 4 °C.

High-molecular-weight (HMW) and low-molecular-weight (LMW) hyaluronic acid levels were quantified using commercially available ELISA kits (FineTest, EU2556 and EU2556-M, Wuhan, China) according to the manufacturer’s instructions. Standard curves were generated and sample concentrations were calculated using Curve Expert 1.4 software. All measurements were performed in accordance with the manufacturer’s protocol.

### 2.13. Cell Viability Assay

Cell viability assay was performed as described [27]. Cells were seeded in 96-well plates at 2 × 10^3^ cells per well and treated with gemcitabine (0.01 µM) or 4-hydroxymethylcoumarin (200 µM) for 72 h. Cell viability was assessed using the MTT (Biosharp, Beijing, China) assay according to the manufacturer’s instructions. Relative viability was calculated as:

$$\mathrm{Cell viability}\left(\%\right)=\frac{A_{\mathrm{treated}}}{A_{\mathrm{control}}}\times 100$$

All experiments were performed in triplicate.

### 2.14. Wound Healing Assay

Experiments were performed as described [28]. CFPAC-1, MIA PaCa-2, and MIA PaCa-2-*CD44*-KO cells were seeded at equal densities in 12-well plates and cultured to 90–100% confluence. A uniform linear wound was created using a sterile 100 µL pipette tip. Cells were washed with PBS to remove detached cells and cultured in serum-free medium.

Cells were assigned to three treatment groups: (1) control (CTR), receiving vehicle only; (2) HA group, treated with hyaluronic acid (HA, 50 µg/mL); and (3) HA+E group, treated with HA (50 µg/mL) and hyaluronidase (10 µg /mL). Images of the same wound area were captured at 0, 4, and 24 h using an inverted microscope.

Each experiment was performed with three biological replicates, and three fields were analyzed per replicate. Wound closure was quantified using ImageJ software, and the relative migration rate was calculated based on the initial wound area at 0 h. Statistical significance was determined using two-way ANOVA.

### 2.15. Transwell Migration and Invasion Assays

Transwell steps were performed as described [29]. Cell migration was assessed using 24-well Falcon^®^ Cell Culture Inserts (Corning, Glendale, AZ, USA). For invasion assays, the upper chamber membranes were coated with 100 µL of Matrigel Basement Membrane Matrix (Corning, No. 354234) diluted 1:8 in coating buffer and incubated for 3 h at 37 °C, following the manufacturer’s instructions. PDAC cells were suspended in 200 µL serum-free DMEM and seeded into the upper chambers (MIA PaCa-2 and MIA PaCa-2-*CD44*-KO, 2 × 10^4^ cells; Pan02, Pan02-*CD44*-sh, CFPAC-1, KPC, 4 × 10^4^ cells). For drug experiment, different cells first exposed to 4-Methylumbelliferone (200 µM) for 72 h, lower chambers contained 500 µL DMEM supplemented with 10% FBS. Cells were incubated for 48 h at 37 °C in a humidified atmosphere with 5% CO_2_. After incubation, cells that had migrated or invaded through the membrane were fixed with paraformaldehyde (Servicebio, Wuhan, China) and stained with crystal violet (Servicebio, Wuhan, China). Cells were subsequently counted under a microscope.

### 2.16. Human Tissue Samples, Immunohistochemistry (IHC) and Immunofluorescence (IF)

Paraffin-embedded pancreatic ductal adenocarcinoma (PDAC) tissues and matched adjacent normal pancreatic tissues were obtained from 28 patients who underwent surgical resection at Wuxi Second People’s Hospital. In addition, PDAC tumor specimens from six patients were collected at Nanjing First Hospital (Nanjing, China).

All procedures were conducted in accordance with the Declaration of Helsinki. Written informed consent was obtained from all participants. The study was approved by the Biobank Ethics Committee of Nanjing First Hospital (Approval No. KY20250527-10) and the Medical Ethics Committee of Wuxi Second People’s Hospital, Affiliated Hospital of Jiangnan University (Approval No. WXEY-2025-211).

For immunohistochemistry (IHC), primary antibodies against CD44 (CST, 37259S, Danvers, MA, USA), p63 (Abways, CY5659, Shanghai, China), and β-catenin (Abways, CY3523, Shanghai, China) were used at a dilution of 1:300. For immunofluorescence (IF) staining of clinical tissue samples, antibodies against CD44 (AiFang Biological, AF20017, Hunan, China) and E-cadherin (AiFang Biological, AF01810, Hunan, China) were used at a dilution of 1:300. For IF staining of cultured cells, antibodies against CD44 (Immunoway, YM4707, San Jose, CA, USA) and E-cadherin (HUABIO, HA723564, Hangzhou, China) were used at a dilution of 1:300.

Images were acquired using a confocal or fluorescence microscope. Detailed procedures for tissue processing, immunohistochemistry, and immunofluorescence staining are provided in the Supplementary Methods.

### 2.17. RNA Sequencing Analysis

Total RNA was extracted from cells using standard protocols and subjected to transcriptome sequencing by commercial service providers. RNA quality was assessed prior to library construction, and sequencing libraries were prepared according to the manufacturers’ protocols. Paired-end sequencing was performed on an Illumina platform.

Raw sequencing data were subjected to quality control and adapter trimming before alignment to the human reference genome. Gene expression levels were quantified and normalized for downstream analyses. Differentially expressed genes (DEGs) were identified using established statistical methods with thresholds of |log2 fold change| > 1 and adjusted *p* < 0.05. Functional enrichment analyses, including Gene Ontology (GO) and Kyoto Encyclopedia of Genes and Genomes (KEGG) pathway analyses, were performed to investigate biological processes and signaling pathways associated with the identified DEGs.

The data generated in this study are publicly available in Gene Expression Omnibus (GEO) at GSE320416 and GSE333917.

Detailed information regarding library preparation, sequencing parameters, quality control procedures, and bioinformatic analyses for each dataset is provided in the Supplementary Methods.

### 2.18. Dataset Analysis

Kaplan–Meier survival curves were generated using the online Kaplan–Meier plotter tool (http://kmplot.com/analysis/) (accessed on 23 March 2026).

RNA sequencing data and matched clinical annotations for 181 pancreatic ductal adenocarcinoma (PDAC) patients (TCGA-PAAD cohort) were retrieved from The Cancer Genome Atlas (TCGA) Data Portal (https://portal.gdc.cancer.gov/, accessed on 19 October 2026). For differential gene expression analysis and functional pathway enrichment, samples were stratified into *CD44*-high and *CD44*-low expression groups based on the upper and lower quartiles of *CD44* mRNA expression. In contrast, for Gene Set Enrichment Analysis (GSEA), binary stratification was performed using the median *CD44* expression level as the threshold. The Progenitor-PDA_identity_signature and Squamous-PDA_identity_signature gene sets—originally defined and validated in the article titled “Cell of Origin Influences Pancreatic Cancer Subtype” [30]—were applied as reference signatures for GSEA.

Gene expression profiles of human pancreatic cancer cell lines were retrieved from the Gene Expression Omnibus (GEO) database (https://www.ncbi.nlm.nih.gov/geo/) (accessed on 7 July 2025) under accession numbers GSE36924 and GSE71729.

Density map analysis was generated by 10x Genomics Xenium Explorer 4.1.0 and the data generated in this study are publicly available in Gene Expression Omnibus (GEO) at GSE274673.

Correlation analysis was generated from Gepia2 (http://gepia2.cancer-pku.cn/#index) (accessed on 9 March 2026) Public Library of Bioinformatics. Gene signatures were listed in Table S2.

### 2.19. Statistical Analysis

Statistical analyses were performed using GraphPad Prism 11. Data are presented as mean ± SD, as indicated in the figure legends. The number of independent biological replicates or experimental units (n) is specified in the figure legends. Technical replicates were not considered independent biological replicates.

For comparisons between two groups, unpaired two-tailed Student’s t-tests were used where appropriate. For comparisons among three or more groups with a single independent factor, one-way analysis of variance (ANOVA) was performed, followed by Tukey’s multiple-comparisons test. For experiments involving two independent factors, two-way ANOVA was performed to assess the main effects of each factor and their interaction, followed by Tukey’s multiple-comparisons test. All tests were two-sided, and *p* < 0.05 was considered statistically significant. *, *p* < 0.05; **, *p* < 0.01; ***, *p* < 0.001; ****, *p* < 0.0001 with a confidence interval of 95%.

## 3. Results

### 3.1. CD44 Expression Is Associated with Aggressive and Basal-like Features of PDAC

**Figure 1:**
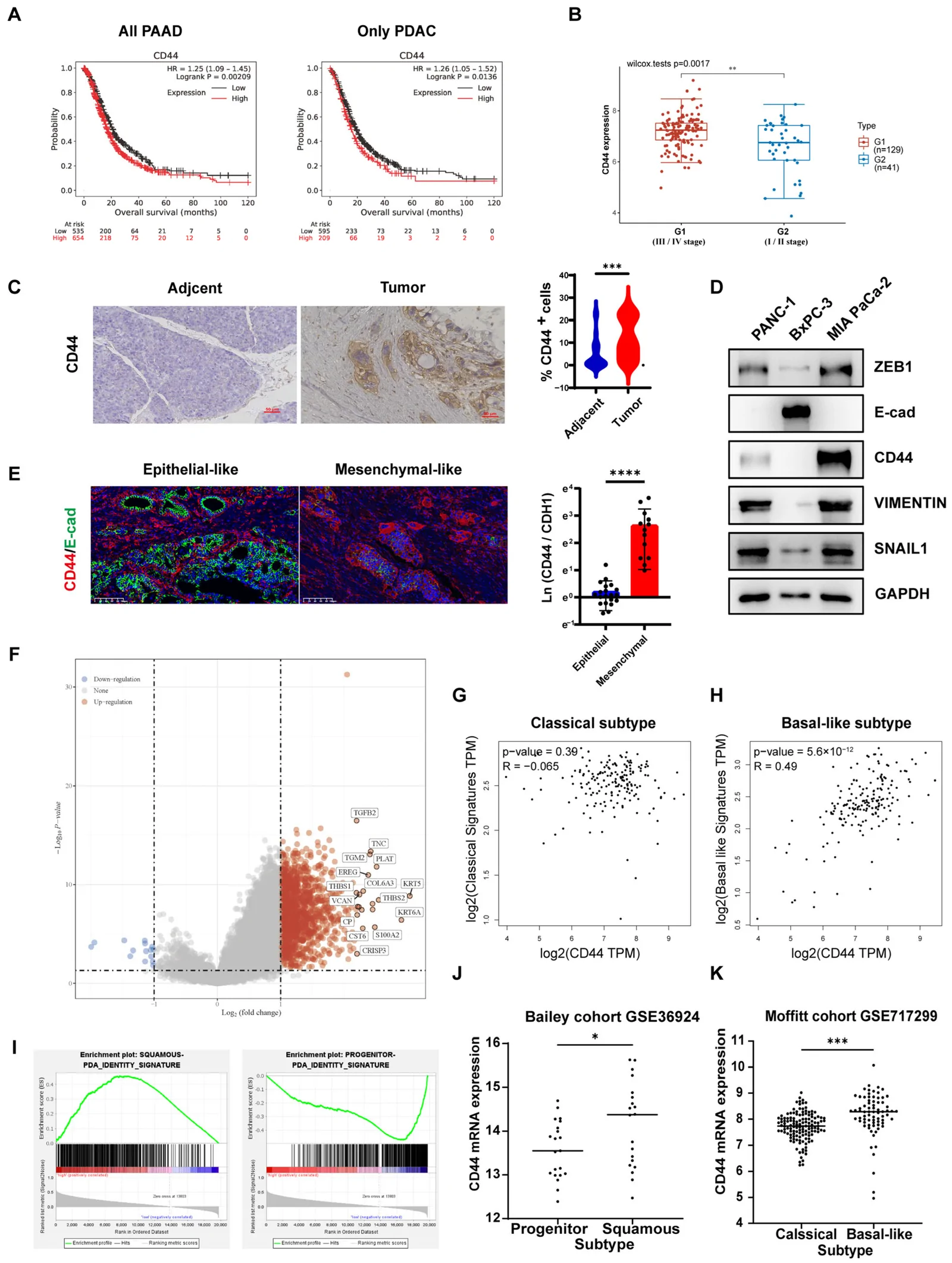
CD44 expression is associated with aggressive and basal-like features of PDAC. (**A**). Analysis of The Cancer Genome Atlas–PDAC/PAAD dataset for associations of *CD44*-high and *CD44*-low expression with overall survival (OS). (**B**). *CD44* mRNA expression by PDAC stage: G1 (n = 129, stage I–II) and G2 (n = 41, stage III–IV) in TCGA PDAC dataset, Wilcox tests, ** *p* < 0.01. (**C**). Representative IHC images and Quantification of CD44-positive cells in tumors and matched adjacent non-tumor tissues (n = 28) in PDAC samples; unpaired Student *t* test, mean ± SD, *** *p* < 0.001. (**D**). Immunoblot analysis of EMT-associated proteins (ZEB1, E-cadherin, vimentin, Snail) and CD44 in BxPC-3, PANC-1, and MIA PaCa-2 cells, GAPDH was used as an internal loading control. (**E**). Representative immunofluorescence images showing CD44 and E-cadherin (E-cad) co-localization and Quantification of CD44/E-cadherin in epithelial-like (n = 20) and mesenchymal-like (n = 13) patient tumor tissue; unpaired Student *t* test, mean ± SD, **** *p* < 0.0001. (**F**). *CD44* differential expression in TCGA PDAC dataset. (**G**). Correlation between *CD44* and the classical associated signature; r and *p* values shown. (**H**). Correlation between *CD44* and the basal-like associated signature; r and *p* values shown. (**I**). GSEA showing the squamous signature and the progenitor signature association in *CD44* up-regulation and down-regulation tumors. (**J**,**K**) *CD44* expression across PDAC molecular subtypes in the Bailey (GSE36924) (Progenitor n = 21, Squamous n = 20) and Moffitt (GSE71729) (classical n = 132, basal-like n = 89) cohorts, unpaired Student *t* test, mean ± SD, * *p* < 0.05, *** *p* < 0.001.

To investigate the role of CD44 in PDAC, we first analyzed the TCGA-PAAD cohort. Elevated *CD44* expression was significantly associated with shorter overall survival and was preferentially associated with poor prognosis in PDAC compared with other gastrointestinal and lung cancers (Figure 1A; Supplementary Figure S1A). Consistently, *CD44* expression was higher in advanced-stage (stage III/IV) than early-stage tumors (Figure 1B). Meanwhile, IHC analysis of 28 clinical PDAC specimens further confirmed markedly higher CD44 expression in tumor tissues than in adjacent non-tumorous tissues (Figure 1C).

We next examined CD44 across pancreatic cancer cell lines. CD44 was nearly undetectable in BxPC-3 cells but highly expressed in MIA PaCa-2 and PANC-1 cells. CD44-high cells exhibited spindle-shaped, mesenchymal-like morphology, increased expression of ZEB1, SNAIL, and Vimentin, and reduced E-cadherin (E-cad) expression compared with CD44-low cells (Figure 1D). Notably, although CD44 was broadly elevated in tumor tissues, its abundance relative to E-cadherin varied substantially across tumor regions (Figure 1E), suggesting that CD44 expression may be linked to changes in epithelial organization during PDAC progression.

We therefore investigated the relationship between *CD44* and PDAC molecular states. In TCGA, *CD44*-high tumors showed enrichment of pro-invasive and basal-like genes, including *COL6A3*, *TGFB2*, *S100A2*, *KRT5*, *KRT6A*, and *TGM2* (Figure 1F). Consistently, CD44-high regions frequently overlapped with TP63-positive areas, particularly in poorly differentiated tumor regions (Supplementary Figure S1B). GEPIA2 analysis further demonstrated that *CD44* expression positively correlated with basal-like, but not classical, gene signatures (Figure 1G,H), and was associated with EMT and poor prognosis related signatures [31] (Supplementary Figure S1C,D). GSEA showed enrichment of classical features in *CD44*-low tumors, whereas *CD44*-high tumors were enriched for *KRAS*, *MYC*, and *TGF-β* signaling pathways (Figure 1I; Supplementary Figure S1E,F). These associations were independently confirmed in GEO datasets classified according to the Moffitt and Bailey subtype systems, in which *CD44* expression was consistently higher in squamous or basal-like tumors (Figure 1J,K).

Collectively, these findings establish a strong association between elevated CD44 expression, PDAC progression, and basal-like features. The marked variation in CD44 abundance relative to E-cadherin further prompted us to investigate whether CD44 undergoes state-dependent changes in its spatial organization and functional role.

### 3.2. The CD44/CDH1 Axis Exhibits State-Dependent Spatial and Quantitative Organization

**Figure 2:**
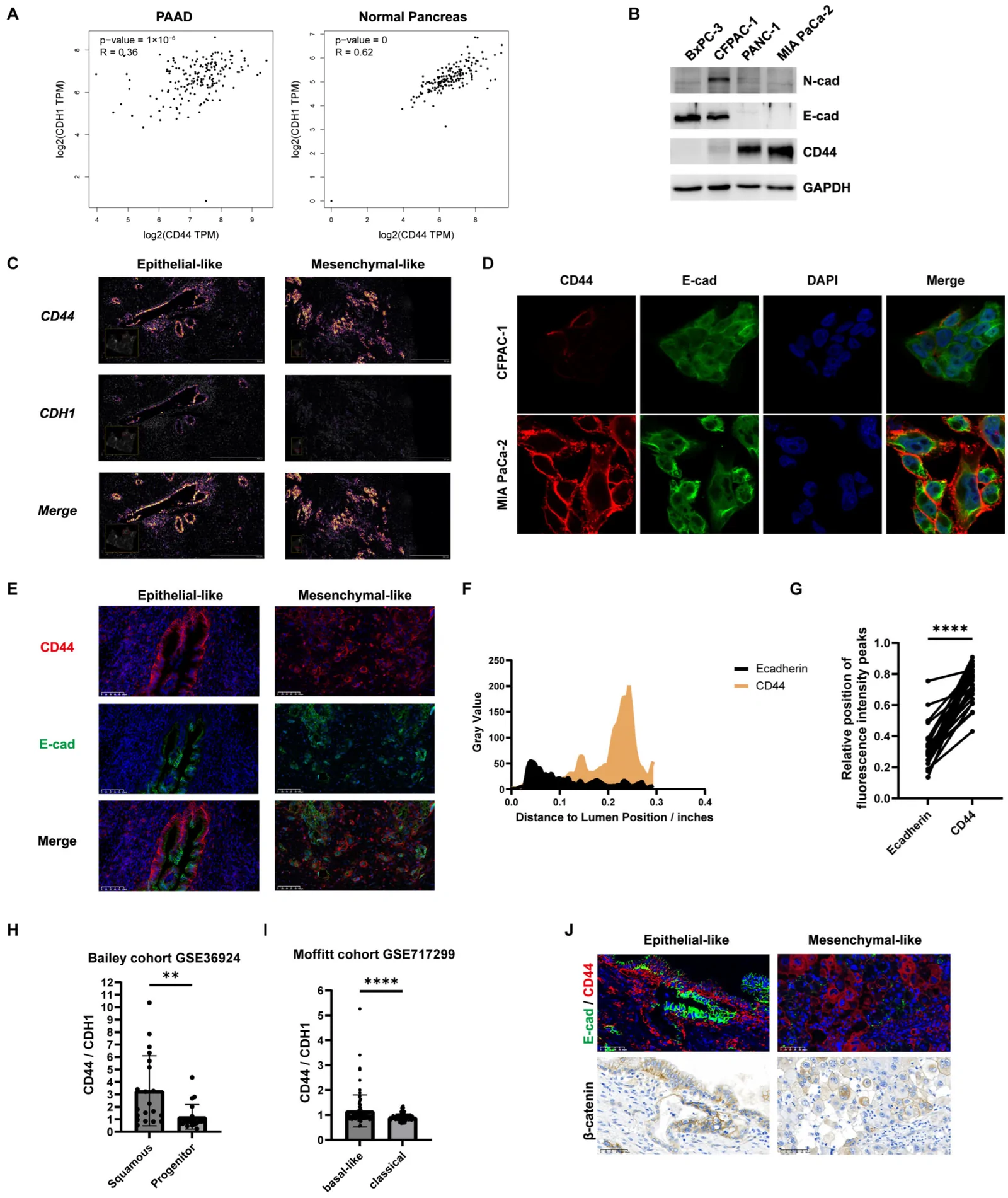
The *CD44/CDH1* axis exhibits state-dependent spatial and quantitative organization (**A**). Correlation between *CD44* and *CDH1* in PAAD and normal pancreas using GEPIA2 platform. (**B**). Immunoblot analysis of EMT-related proteins (E-cadherin, N-cadherin, CD44, GAPDH) in BxPC-3, CFPAC-1, PANC-1, and MIA PaCa-2 cells. (**C**). Representative spatial density maps showing *CD44* and *CDH1* transcript distribution in epithelial-like and mesenchymal-like patient tumor tissue using 10x Genomics from GEO datasets (GSE274673). (**D**). Representative immunofluorescence images showing co-localization of CD44 and E-cadherin (E-cad) in classical subtype CFPAC-1 cells and basal-like subtype MIA-PaCa-2 cells. (**E**). Representative co-localization images of CD44 and E-cadherin (E-cad) in epithelial-like and mesenchymal-like patient tumor tissue from PDAC patients. (**F**). Quantification of co-localization immunofluorescence of CD44 and E-cadherin in epithelial-like tumor samples. (**G**). Quantification of the localization difference between the fluorescence intensity peaks of CD44 and E-cadherin in 27 epithelial-like tumor tissue regions, unpaired Student *t* test, n = 27, mean ± SD, **** *p* < 0.0001. (**H**,**I**). *CD44/CDH1* across PDAC molecular subtypes in the Bailey (GSE36924) (Progenitor n = 21, Squamous n = 20) and Moffitt (GSE71729) (classical n = 132, basal-like n = 89) cohorts, unpaired Student *t* test, mean ± SD, ** *p* < 0.01, **** *p* < 0.0001. (**J**). Representative immunofluorescence co-localization images of CD44 and E-cadherin and IHC for β-catenin in epithelial and mesenchymal PDAC tumor tissues.

Given the association of CD44 with E-cadherin and EMT observed above, we next examined their quantitative and spatial relationship across PDAC states. Analysis of the GEPIA2 dataset showed a modest positive correlation between *CD44* and *CDH1* expression in PDAC tumors, which was stronger in adjacent normal tissues (Figure 2A). In contrast, protein analysis across pancreatic cancer cell lines revealed an inverse relationship between CD44 and E-cadherin expression (Figure 2B), suggesting that their relationship may change with tumor differentiation state.

Analysis of clinical specimens revealed distinct CD44 and E-cadherin patterns according to tumor differentiation (Supplementary Figure S2A). Poorly differentiated tumors exhibited high and diffuse CD44 expression accompanied by reduced E-cadherin, whereas well-differentiated tumors showed lower CD44 expression with preserved E-cadherin. Public transcriptomic datasets showed a similar quantitative relationship, with *CD44* increased and *CDH1* decreased in poorly differentiated tumors (Figure 2C). Importantly, multiplex immunofluorescence in both cells and tumor tissues revealed that CD44 and E-cadherin were not simply inversely expressed in well-differentiated epithelial-like cells; rather, they occupied distinct membrane domains. CD44 was enriched along the basolateral membrane, whereas E-cadherin was concentrated at cell–cell junctions (Figure 2D–G). Thus, their relationship was characterized primarily by spatial segregation in differentiated cells, but by quantitative imbalance in poorly differentiated cells.

Consistent with this distinction, the *CD44/CDH1* ratio remained relatively close to 1 in differentiated tumors but was markedly increased in poorly differentiated tumors (Figure 1E). Independent validation in the Bailey and Moffitt datasets confirmed a similar increase in the *CD44/CDH1* ratio in basal-like tumors (Figure 2H,I). Consistent with these findings, survival analysis suggested that high *CD44* expression was associated with a relatively favorable outcome in well-differentiated PDAC, whereas the opposite trend was observed in poorly differentiated tumors, although these differences did not reach statistical significance (Supplementary Figure S2B). These findings suggest that the combined quantitative relationship between *CD44* and *CDH1* may provide a more informative indicator of PDAC cellular state than CD44 expression alone.

We next examined whether this altered *CD44/CDH1* relationship was accompanied by changes in extracellular matrix interactions and epithelial polarity. CD44-associated matrix signatures differed across PDAC states, with FN1 enriched in mesenchymal-like tumors, whereas basement membrane components including LAMA2 and LAMC3 were preferentially associated with classical tumors (Supplementary Figure S2C–F). Consistently, polarity-related gene signatures and *CTNNB1* expression were associated with *CD44* (Supplementary Figure S2G–I). β-Catenin was predominantly localized to the plasma membrane and cell junctions in differentiated tumors but showed reduced membranous and increased cytoplasmic localization in poorly differentiated tumors (Figure 2J; Supplementary Figure S2J).

Together, these findings identify a state-dependent reorganization of the *CD44/CDH1* axis: *CD44* and *CDH1* are spatially compartmentalized in differentiated PDAC, whereas increased *CD44* and loss of *CDH1* accompany the disorganization of epithelial architecture in poorly differentiated and basal-like states.

### 3.3. CD44 Depletion Induces Distinct Transcriptional Responses Across PDAC States

**Figure 3:**
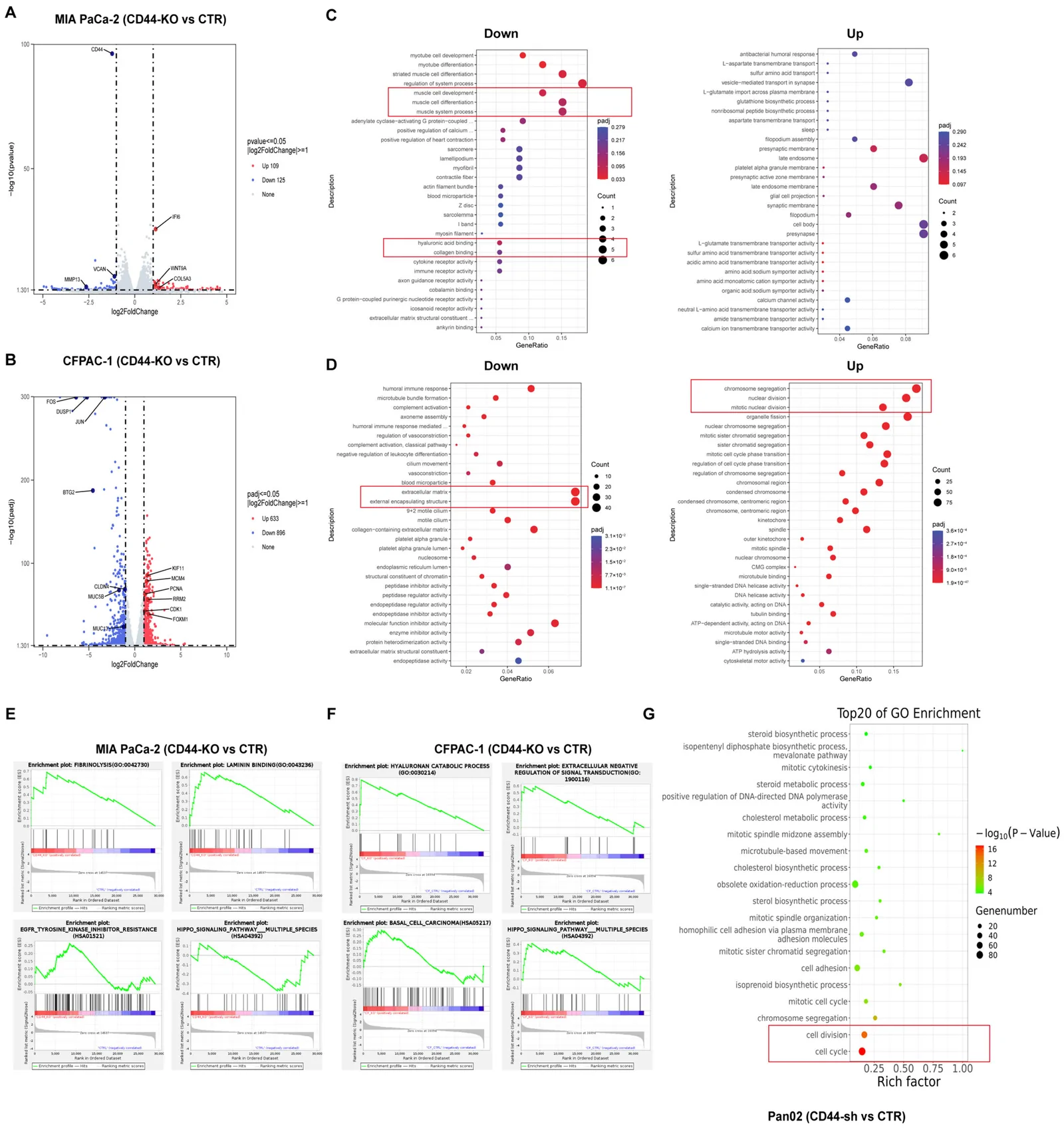
*CD44* depletion induces distinct transcriptional responses across PDAC states (**A**). Differential gene expression analysis between CTR and *CD44*-KO groups in MIA PaCa-2 cells. (**B**). Differential gene expression analysis between CTR and *CD44*-KO groups in CFPAC-1 cells. (**C**). GO Enrichment of CTR and *CD44*-KO groups in MIA PACA-2 cells. (**D**). GO Enrichment of CTR and *CD44*-KO groups in CFPAC-1 cells. (**E**). GSEA of CTR and *CD44*-KO groups on FIBRINOLYSIS, LAMININ BINDING, EGFR-TYROSINE-KINASE-INHIBITOR-RESISTANCE and HIPPO-PATHWAY in MIA PACA-2 cells. (**F**). GSEA of CTR and *CD44*-KO groups on HTALURONAN-CATABOLIC-PROCESS, EXTRACELLULAR NEGATIVE REGULATION OF SIGNAL TRANSDUCTION, BASAL-CELL-CARCINOMA and HIPPO-PATHWAY in CFPAC-1 cells. (**G**). GO Enrichment of CTR and *CD44*-sh groups in Pan02 cells.

To determine whether the functional consequences of *CD44* loss differ across PDAC states, we generated *CD44*-knockout (*CD44*-KO) CFPAC-1 and MIA PaCa-2 cells using CRISPR/Cas9 and performed RNA sequencing. *CD44* depletion produced markedly different transcriptional responses between the two models, with 1529 differentially expressed genes (DEGs) in CFPAC-1 cells compared with 234 in MIA PaCa-2 cells (Figure 3A,B). In MIA PaCa-2 cells, *CD44* loss predominantly affected extracellular matrix-associated genes, including *VCAN* and *MMP13*. In contrast, CFPAC-1 cells showed broader transcriptional changes involving *AP-1* factors (*FOS*, *JUN*, and *DUSP1*), epithelial-associated genes (*CLDN4*, *MUC13*, *MUC5B*, and *BTG2*), and cell-cycle regulators including *MCM4*, *RRM2*, *CDK1*, *FOXM1*, and *PCNA* (Figure 3A,B).

Pathway analyses further revealed state-dependent responses to *CD44* depletion. In MIA PaCa-2 cells, *CD44* loss altered extracellular matrix remodeling, laminin interaction, cytoskeletal organization, and contractile programs, consistent with changes in invasive phenotypes (Figure 3C,E; Supplementary Figure S3A). In CFPAC-1 cells, the affected pathways were primarily related to protease activity, extracellular matrix remodeling, and cell proliferation (Figure 3D,F; Supplementary Figure S3B). Notably, *HIPPO* pathway activity showed opposite changes following *CD44* depletion between the two models, further supporting distinct transcriptional consequences of *CD44* loss across PDAC states (Figure 3E,F).

To extend these findings to another basal-like model, we depleted *CD44* in murine Pan02 cells. *CD44* loss reduced cell proliferation and was accompanied by changes in *WNT*, *HIPPO*, and *TGF-β* signaling pathways (Figure 3G; Supplementary Figure S3C). Together, these transcriptomic analyses demonstrate that *CD44* depletion elicits state-dependent transcriptional responses, with basal-like models showing prominent alterations in extracellular matrix and malignant-associated programs, whereas classical cells undergo broader changes involving epithelial and proliferative programs.

### 3.4. CD44 Preferentially Promotes Malignant Phenotypes in Basal-like PDAC

**Figure 4:**
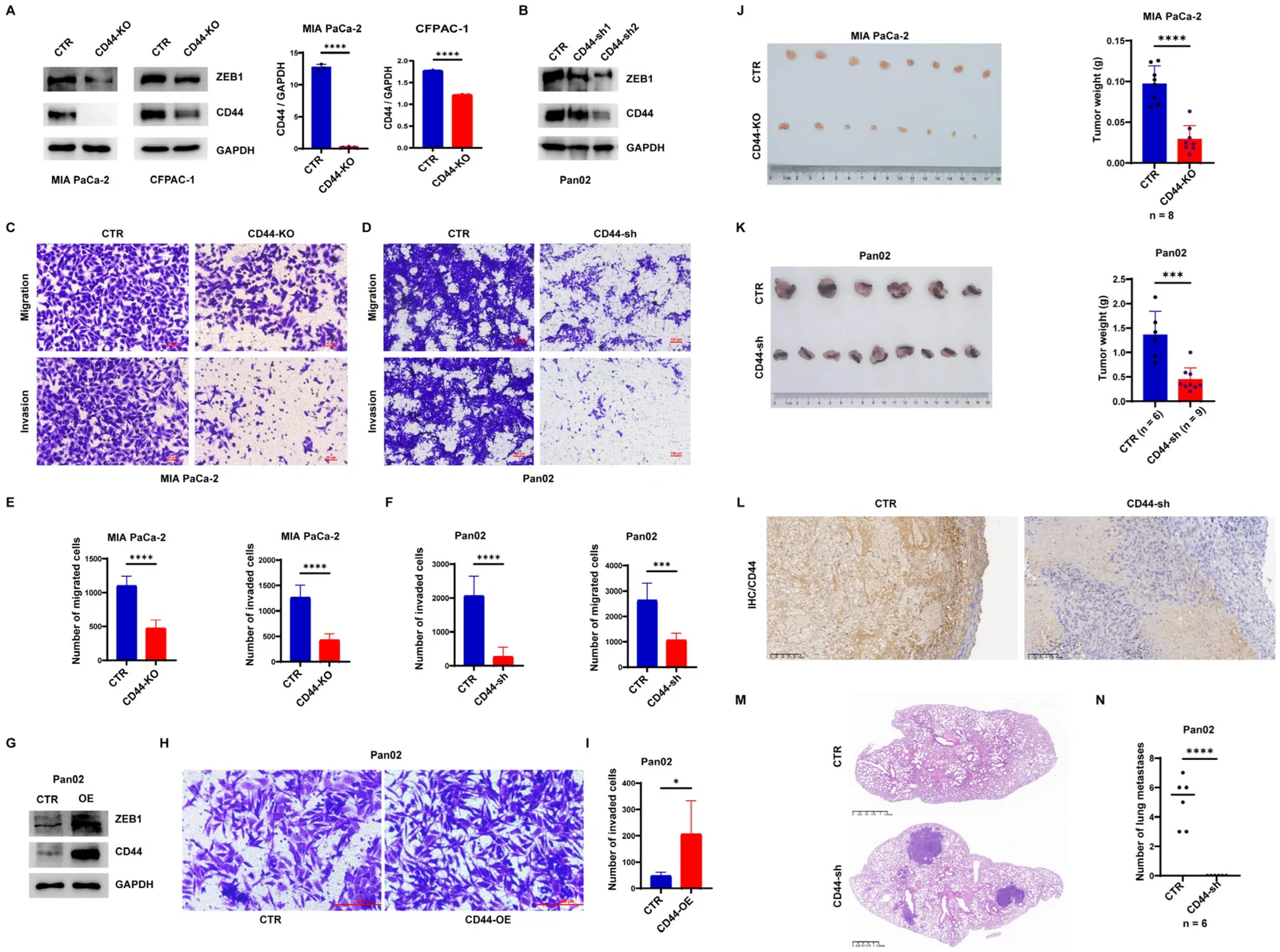
CD44 preferentially promotes malignant phenotypes in basal-like PDAC (**A**). Immunoblot analysis of CD44, ZEB1 and E-cad expression in CTR and *CD44*-KO groups in MIA PACA-2 and CFPAC-1 cells, GAPDH was used as an internal loading control. Data represent mean ± SD for at least three independent experiments; unpaired Student *t* test, **** *p* < 0.0001 (**B**). Immunoblot analysis of CD44, ZEB1 and GAPDH expression in CTR and *CD44*-sh groups in Pan02 cells. (**C**–**F**). Migration and invasion assays showing reduced motility in MIA PACA-2 (**C**,**E**) and Pan02 (**D**,**F**) cells with *CD44*-KO in MIA PaCa-2 and *CD44*-sh in Pan02 compared with vector controls. Data represent mean ± SD for at least five independent experiments; unpaired Student *t* test, **** *p* < 0.0001, *** *p* < 0.001. (**G**). Immunoblot analysis of CD44, E-cadherin, and ZEB1 in Pan02 cells following *CD44* overexpression. (**H**,**I**). *CD44*-overexpressing Pan02 cells exhibited increased invasion compared with vector controls. Data represent mean ± SD for at least three independent experiments; unpaired Student *t* test, * *p* < 0.05. (**J**). Representative images and tumor weights of subcutaneous xenograft tumors derived from MIA PaCa-2 wild-type (CTR) cells and *CD44*-knockout (*CD44*-KO) cells, n = 8, unpaired Student *t* test, mean ± SD, **** *p* < 0.0001. (**K**). Representative images and tumor weights of orthotopic pancreatic xenograft tumors derived from Pan02 wild-type (CTR) cells and *CD44*-sh cells, n(CTR) = 6, n(*CD44*-sh) = 9, unpaired Student *t* test, mean ± SD, *** *p* < 0.001. (**L**). Representative immunohistochemistry (IHC) images showing CD44 expression in tumors derived from Pan02 CTR and *CD44*-sh cells. (**M**). Representative hematoxylin and eosin (H&E) staining images of lung metastatic lesions (n = 6). (**N**). quantification of metastatic nodules, unpaired Student *t* test, mean ± SD, **** *p* < 0.0001.

We next asked whether the state-dependent transcriptional responses to CD44 depletion were reflected at the protein and phenotypic levels. *CD44* knockout reduced ZEB1 expression across the tested cell lines, with the strongest effect observed in MIA PaCa-2 cells. In these cells, CD44 loss additionally reduced SNAIL, COL1A1, and TGFβR expression, whereas the protein profile of CFPAC-1 cells was largely unchanged (Figure 4A,B; Supplementary Figure S4A,B). Consistently, CD44 depletion markedly reduced migration and invasion in MIA PaCa-2 and Pan02 cells, but not in CFPAC-1 cells (Figure 4C–F; Supplementary Figure S4C). Conversely, CD44 overexpression increased ZEB1 expression in PANC-1 and Pan02 cells and enhanced the invasive capacity of Pan02 cells (Figure 4G–I; Supplementary Figure S4D).

We next assessed the effects of CD44 depletion in vivo. *CD44* knockout significantly reduced tumor growth in a MIA PaCa-2 subcutaneous xenograft model (Figure 4J). In an orthotopic Pan02 model, *CD44* knockdown reduced primary tumor burden and markedly suppressed peritoneal dissemination (Figure 4K,L). Similarly, in a tail-vein metastasis model, *CD44*-depleted cells produced substantially fewer pulmonary metastatic lesions than control cells (Figure 4M,N). Together, these findings demonstrate that CD44 makes a greater functional contribution to EMT-associated phenotypes, tumor growth, and metastatic dissemination in basal-like PDAC models.

### 3.5. Basal-like PDAC Cells Exhibit Heightened Sensitivity to HA–CD44 Signaling

**Figure 5:**
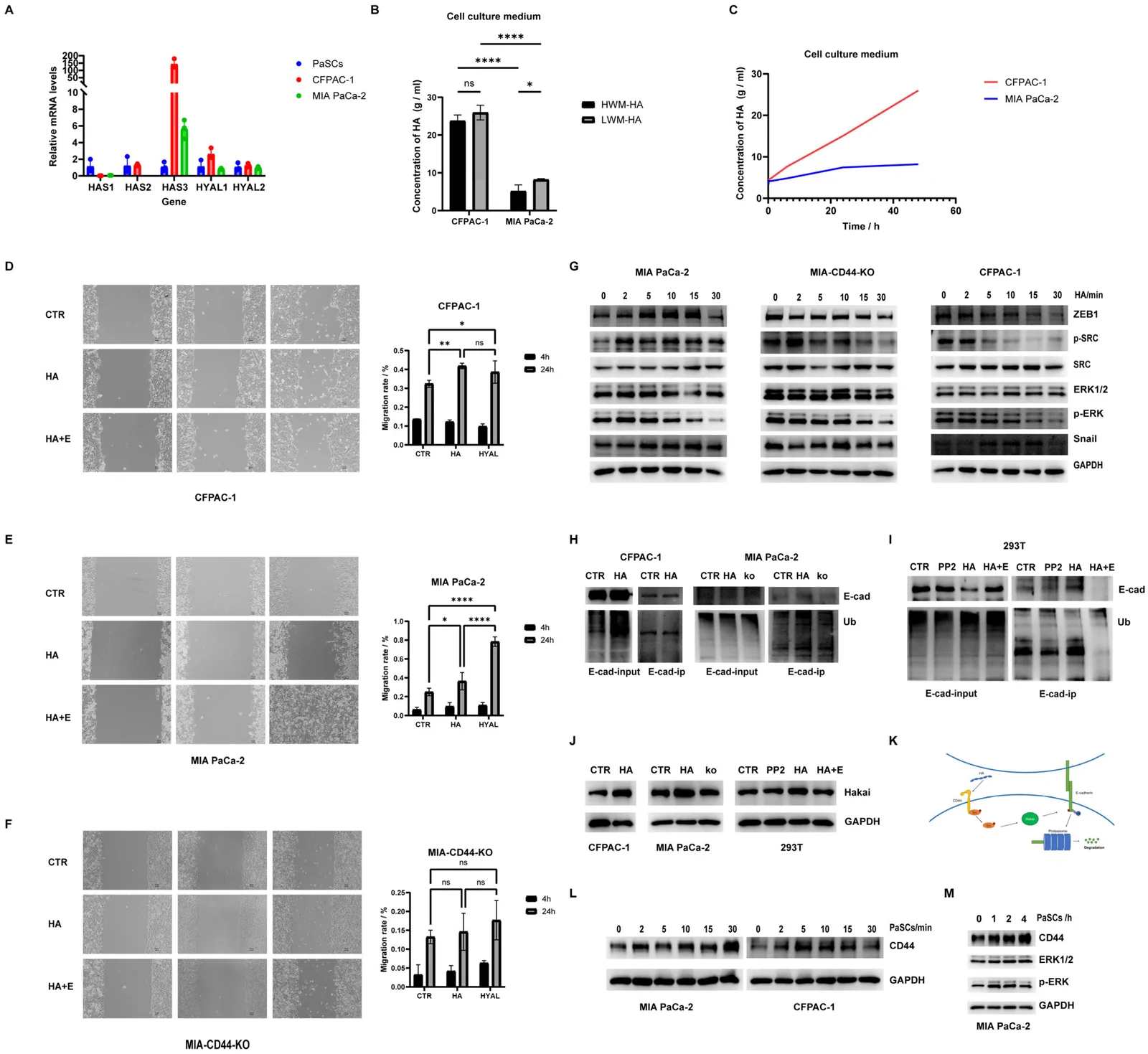
Basal-like PDAC cells exhibit heightened sensitivity to HA–CD44 signaling (**A**)**.** Quantification of *HAS1*, *HAS2*, *HAS3*, *HYAL1* and *HYAL2* mRNA expression in PaSCs, CFPAC-1 and MIA PaCa-2 cells. (**B**). High-molecular-HA and Low-molecular-HA levels in the supernatants of CFPAC-1 and MIA PaCa-2 cells measured by ELISA, two-way ANOVA, followed by Tukey’s multiple-comparisons test, mean ± SD, ns, no significant, * *p* < 0.05, **** *p* < 0.0001. (**C**). ELISA analysis of the time-dependent changes in low-molecular-weight hyaluronic acid concentration in the supernatants of CFPAC-1 and MIA PaCa-2 cells after addition of fresh medium. (**D–F**). Typical images and quantitative results of the scratch assay for CFPAC-1 (**D**), MIA PaCa-2 (**E**) and MIA-*CD44*-KO (**F**) cells in different groups (CTR = CTRL, HA = hyaluronic acid, HA+E = hyaluronic acid + hyaluronidase) (HA: 50 µg/mL) (hyaluronidase: 10 µg /mL). two-way ANOVA, followed by Tukey’s multiple-comparisons test, mean ± SD, ns, no significant, * *p* < 0.05, ** *p* < 0.01, **** *p* < 0.0001. (**G**). Immunoblot analysis of time-course protein expression (0, 2, 5, 10, 15, and 30 min) following pretreatment with PP2 (10 µM), a SRC inhibitor, and stimulation with hyaluronic acid (HA: 50 µg/mL) in MIA-PACA-2, CFPAC-1, and MIA-*CD44*-KO cells. Target proteins included ZEB1, SNAIL, SRC, phosphorylated SRC (p-SRC), ERK, phosphorylated ERK (p-ERK), and the loading control GAPDH. (**H**). E-cadherin ubiquitination in MIA PaCa-2, ko (*CD44*-KO) and CFPAC-1 cells under the HA (50 µg/ml) treatments. (**I**). E-cadherin ubiquitination in 293T cells under the hyaluronic acid (HA) treatments including CTR, PP2 (10 µM), HA (50 µg /ml), HA (50 µg /mL) + hyaluronidase (10 µg /mL). (**J**). Immunoblot analysis of Hakai expression in different cells under the HA treatments. (**K**). Proposed model: HA–CD44 interaction activates SRC, recruits Hakai, and promotes E-cadherin ubiquitination. (**L**). Immunoblot analysis of CD44 protein expression of time-course protein expression (0, 2, 5, 10, 15, and 30 min) following stimulation with PaSCs cell supernatant in MIA PaCa-2 and CFPAC-1 cells. (**M**). Immunoblot analysis of CD44 protein expression of time-course protein expression (0, 1, 2 and 4 h) following stimulation with PaSCs cell supernatant in MIA PaCa-2 cells.

We next investigated whether the interaction between CD44 and its ligand HA differs across PDAC states. High expression of the HA synthesis genes *HAS2* and *HAS3* was associated with poor prognosis, whereas increased expression of the HA-degrading genes *HYAL1* and *HYAL2* showed the opposite trend (Supplementary Figure S5A). Analysis of HA-related genes revealed distinct metabolic profiles between CFPAC-1 and MIA PaCa-2 cells. CFPAC-1 cells expressed both HA synthesis and degradation genes and secreted substantially higher levels of HA, whereas MIA PaCa-2 cells showed lower HA production and relatively higher expression of HA-degrading genes (Figure 5A). To determine whether these transcriptional differences affected HA production, we quantified HA levels in conditioned media by ELISA. CFPAC-1 cells secreted substantially higher levels of HA than MIA PaCa-2 cells (Figure 5B,C). These findings suggested that basal-like cells may be less dependent on cell-autonomous HA production but more responsive to extracellular HA.

We therefore examined cellular responses to exogenous HA. HA promoted migration in both models, but MIA PaCa-2 cells showed a markedly greater response to HA degradation products, as indicated by their enhanced migration following hyaluronidase treatment (Figure 5D,E, Supplementary Figure S7B). Importantly, this response was substantially reduced following *CD44* depletion, supporting a CD44-dependent effect of HA signaling (Figure 5F).

To define the underlying signaling mechanism, we stimulated cells with HA and monitored downstream responses. HA rapidly induced SRC and ERK phosphorylation in MIA PaCa-2 cells, followed by increased ZEB1 and SNAIL expression. These responses were markedly attenuated in *CD44*-deficient cells (Figure 5G, Supplementary Figure S5B). HA stimulation also increased E-cadherin ubiquitination in MIA PaCa-2 and CFPAC-1 cells (Figure 5H). The ubiquitination response was further confirmed in HEK-293T cells (Figure 5I). Moreover, HAKAI expression was upregulated in HA-stimulated groups (Figure 5J; Supplementary Figure S5C). Collectively, HA stimulation activated CD44/SRC signaling and was associated with reduced E-cadherin expression, accompanied by changes in HAKAI-related signaling. These findings suggest that HAKAI may contribute to the regulation of E-cadherin downstream of HA–CD44–SRC signaling (Figure 5K).

Finally, to assess whether stromal signals could differentially regulate CD44, we treated both models with conditioned medium from pancreatic stellate cells. CD44 induction was transient in CFPAC-1 cells but sustained in MIA PaCa-2 cells (Figure 5L,M, Supplementary Figure S5D,E).

Together, these findings reveal a key state-dependent difference in HA–CD44 signaling: although differentiated/classical cells produce more HA, basal-like cells are more sensitive to extracellular HA and its bioactive degradation products. This heightened CD44-dependent responsiveness activates SRC/ERK signaling and promotes EMT-associated changes, providing a potential explanation for the greater functional dependence of basal-like PDAC on CD44.

### 3.6. Targeting the HA–CD44 Axis Sensitizes Basal-like PDAC to Chemotherapy

**Figure 6:**
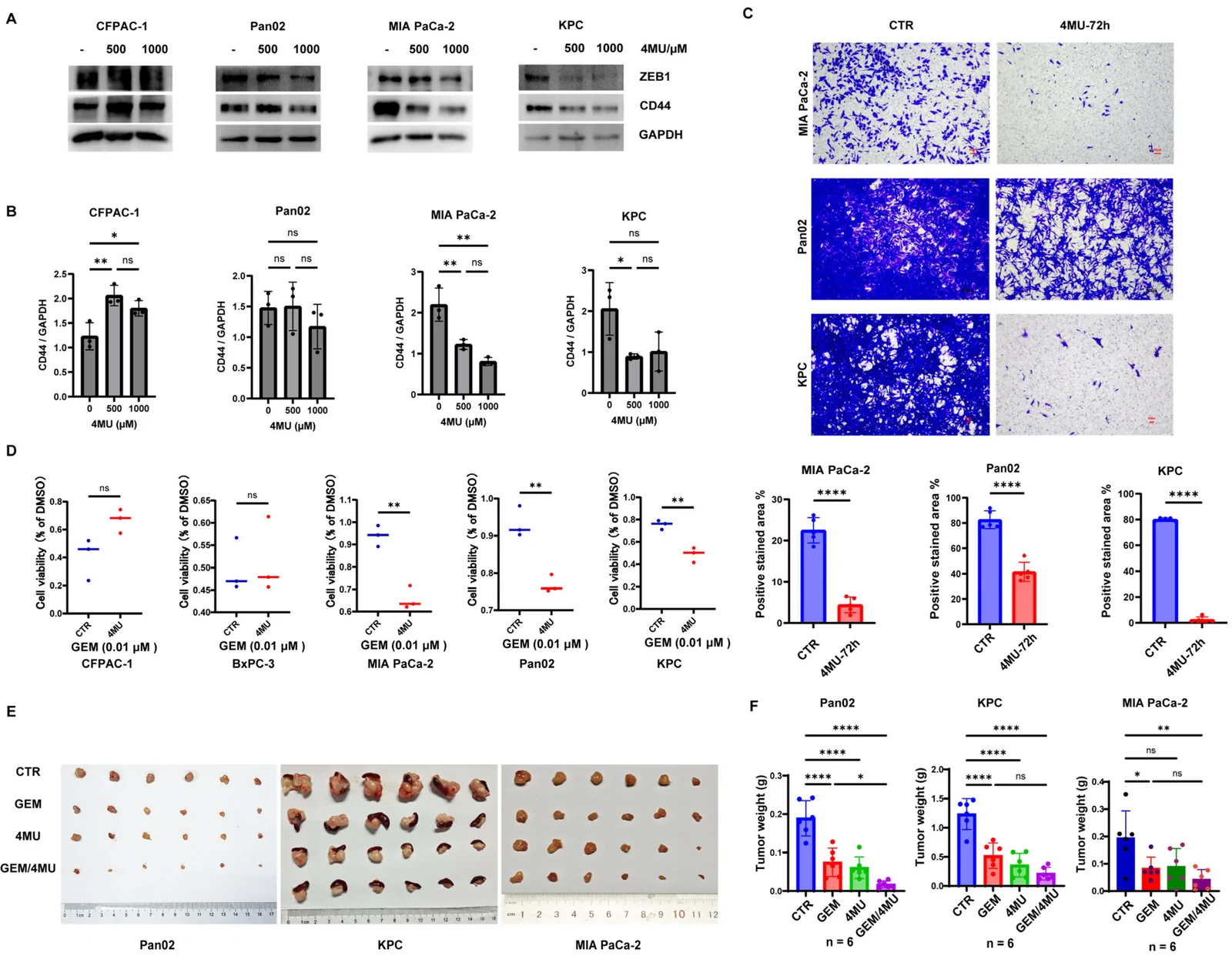
Targeting the HA–CD44 axis sensitizes basal-like PDAC to chemotherapy (**A**). Immunoblot analysis of CD44, ZEB1 and GAPDH in Pan02, MIA PaCa-2, CFPAC-1 and KPC cells treated with different concentrations (500, 1000) of 4-Methylumbelliferone (4MU/µM), an HA synthesis inhibitor. (**B**). Quantification analysis of CD44, ZEB1 and GAPDH in Pan02, MIA PaCa-2, CFPAC-1 and KPC cells treated with different concentrations (500, 1000) of 4-Methylumbelliferone (4MU/µM). one-way ANOVA, followed by Tukey’s multiple-comparisons test, mean ± SD, ns, no significant, * *p* < 0.05, ** *p* < 0.01. (**C**). Representative images and quantification of migration assays showing reduced motility in MIA PaCa-2, Pan02, CFPAC-1 and KPC cells exposed to 4MU (200 µM, 72 h) compared with vector controls, Data represent mean ± SD for at least five independent experiments; Unpaired Student *t* test, **** *p* < 0.0001. (**D**). MTT assay showing the effects of 4MU (200 µM) combined with gemcitabine (GEM/0.01 µM) on CFPAC-1, BxPC-3, Pan02, MIA PaCa-2, and KPC cells. Data represent mean ± SD for at least three independent experiments; unpaired Student *t* test, ns, no significant, ** *p* < 0.01. (**E**). Representative images of orthotopic Pan02, KPC and MIA PaCa-2 tumors from each treatment group, CTR (control), GEM (gemcitabine/) (10 mg/kg), 4MU (4-Methylumbelliferone) (100 mg/kg), GEM/4MU (combined group). (**F**). Quantification of tumor weights corresponding to Pan02, KPC and MIA PaCa-2 orthotopic models, one-way ANOVA, followed by Tukey’s multiple-comparisons test, mean ± SD, ns, no significant, * *p* < 0.05, ** *p* < 0.01, **** *p* < 0.0001.

Given the heightened HA–CD44 responsiveness of basal-like PDAC cells, we next tested whether HA synthesis inhibition could be therapeutically exploited. Treatment with 4-methylumbelliferone (4-MU), an inhibitor of HA synthesis, reduced CD44 and ZEB1 expression and inhibited cell migration, with stronger effects observed in basal-like models, particularly KPC cells (Figure 6A–C).

We next combined 4-MU with gemcitabine (GEM). At a low concentration of GEM, the addition of 4-MU further increased the inhibitory effect of GEM on basal-like MIA PaCa-2, Pan02, and KPC cells, whereas this effect was less pronounced in classical-like CFPAC-1 and BxPC-3 cells (Figure 6D, Supplementary Figure S7A,C). Consistently, combined 4-MU and GEM treatment tended to reduce tumor burden in orthotopic MIA PaCa-2, Pan02 and KPC models, with a significant effect observed in Pan02 tumors (Figure 6E,F; Supplementary Figure S6A). The combination was generally well tolerated, with no evident hepatic toxicity or significant body-weight loss (Supplementary Figure S6B–H).

Together, these findings demonstrate that pharmacological inhibition of HA synthesis preferentially disrupts the CD44-dependent vulnerability of basal-like PDAC and enhances its response to gemcitabine, supporting the HA–CD44 axis as a potential state-selective therapeutic target.

## 4. Discussions

PDAC is characterized by remarkable molecular heterogeneity and plasticity, which contribute to disease progression, therapeutic resistance, and poor clinical outcomes. In this study, we demonstrate that CD44 is associated with aggressive disease and is strongly enriched in the basal-like subtype of PDAC. More importantly, our findings suggest that the biological role of CD44 is highly context-dependent and differs substantially between classical and basal-like tumors.

Consistent with previous studies, elevated CD44 expression was associated with EMT features, aggressive tumor behavior, and unfavorable prognosis [15,32]. However, our data indicate that the biological significance of CD44 extends beyond expression level alone. We observed distinct spatial relationships between CD44 and E-cadherin across different differentiation states. In differentiated and classical tumors, CD44 was predominantly localized to the basolateral membrane, whereas E-cadherin was concentrated at cell–cell junctions. In poorly differentiated and basal-like tumors, increased and redistributed CD44 accompanied marked E-cadherin loss. CD44 was also more closely associated with fibronectin in basal-like cells, whereas laminin family components predominated in classical cells. These findings suggest that the functional context of CD44 varies across PDAC states, with differentiated cells showing an anchoring-associated localization at the cell–matrix interface, whereas basal-like cells exhibit greater HA-responsive signaling and matrix interactions. Although CD44 does not appear to directly replace E-cadherin-mediated adhesion, its redistribution may contribute to the acquisition of migratory and invasive properties. This context-dependent behavior may also offer one explanation for the frequent association of CD44 with cancer stem-like populations, in which cellular plasticity and migratory capacity are prominent features. In all, the spatial and quantitative relationship between *CD44* and *CDH1* may therefore provide additional information for PDAC state stratification beyond CD44 expression alone.

EMT is closely associated with tumor dedifferentiation and the basal-like phenotype in pancreatic ductal adenocarcinoma (PDAC). Previous studies have implicated several mechanisms in EMT regulation, including TGF-β signaling and adaptive responses to chemotherapy. TGF-β activation can induce EMT [33] and promote invasive phenotypes in PDAC [34], whereas TGF-β inhibition has been reported to favor a more classical-like state, highlighting the association between TGF-β signaling and basal-like features [35]. CD44 has also been linked to poor differentiation and cancer stem cell properties [36], and has been implicated in EMT and pancreatic cancer progression [15,37]. Consistent with these findings, our study revealed elevated CD44 expression alongside increased TGF-β and the EMT-associated marker ZEB1 in basal-like PDAC models. In addition, chemotherapy-resistant pancreatic cancer cells have been reported to exhibit increased CD44 expression and enhanced stem-like and mesenchymal features [16,38]. Together, these findings support a close association between CD44 enrichment, EMT-related programs, and aggressive PDAC states, consistent with our analyses of cell models, clinical specimens, and public datasets.

A major finding of this study is the differential responsiveness of CD44 to HA across PDAC states. Although HA is the principal ligand of CD44, its biological effects depend on cellular context. classical cells produced higher levels of HA but showed relatively stable responses to HA stimulation, whereas basal-like cells were more sensitive to HA despite lower overall HA abundance. HA rapidly activated SRC, ERK and EMT signaling to enhance migratory behavior in basal-like cells in a CD44-dependent manner, suggesting that the functional output of HA is determined not simply by ligand abundance but by the signaling state of its receptor. Previous studies showed that HA fragments of different molecular weights exert distinct biological effects [39,40]. High-molecular-weight HA generally supports tissue homeostasis, whereas low-molecular-weight HA promotes inflammation and tumor progression [41,42]. Our findings raise the possibility that the biological consequences of HA signaling depend not only on HA abundance and molecular size but also on the cellular state of the tumor. Although basal-like PDAC cells produced less HA than differentiated cells, they exhibited markedly greater responsiveness to extracellular HA through CD44, suggesting that the same extracellular ligand may elicit distinct biological outputs depending on the state of the responding tumor cell. Thus, HA-mediated tumor progression may be shaped by a dynamic interaction between ligand properties and cellular context, rather than being determined solely by the biochemical properties of HA itself. Meanwhile, this context-dependent HA response may also help explain why broadly depleting stromal HA has produced limited clinical benefit in PDAC [43,44].

From a therapeutic perspective, inhibition of HA synthesis reduced CD44 expression, suppressed malignant phenotypes, and enhanced gemcitabine sensitivity, particularly in basal-like models. These preliminary findings suggest that 4-MU may enhance the inhibitory effect of gemcitabine in basal-like PDAC cells. However, this effect cannot be attributed exclusively to the HA–CD44 axis, as HA-independent effects of 4-MU may also contribute. Moreover, in the absence of formal drug-interaction analysis, whether the combined effect is synergistic or additive remains unclear. Further studies are needed to clarify the mechanisms underlying this combination effect. These findings identify the HA–CD44 axis as a potential state-selective therapeutic vulnerability in aggressive PDAC. The CD44/CDH1 spatial-quantitative relationship may also provide a histopathological feature complementary to current molecular classification approaches. Importantly, our results suggest that therapeutic targeting of CD44-related pathways may require consideration of both target abundance and cellular context.

Several limitations should be acknowledged. First, our study primarily examined the standard CD44 isoform (CD44s) and did not systematically distinguish the functional contributions of alternative CD44 splice variants. Given that CD44 isoforms may exhibit distinct ligand interactions, subcellular localization, and signaling properties, future studies should determine whether isoform-specific regulation contributes to the context-dependent behavior observed here [45]. In addition, although our clinical specimens were characterized according to pathological differentiation and stage, transcriptomic subtype assignment was not available for all samples. Future studies integrating spatial or single-cell transcriptomic profiling with CD44 localization will be important to further resolve the relationship between CD44 spatial organization and dynamic PDAC cellular states.

## 5. Conclusions

In conclusion, our study demonstrates that the biological roles of CD44 vary across pancreatic ductal adenocarcinoma states and are associated with differences in its spatial organization. In differentiated PDAC, CD44 was predominantly localized to the basolateral membrane and spatially segregated from E-cadherin at cell–cell junctions, consistent with a role in epithelial organization and cell–matrix interactions. In poorly differentiated and basal-like PDAC, CD44 exhibited increased abundance and redistribution along the cell periphery, accompanied by enhanced hyaluronan responsiveness and malignant phenotypes. These findings suggest that the functional output of CD44 is influenced not only by its expression level but also by cellular context and spatial organization. Inhibition of hyaluronan synthesis preferentially suppressed basal-like PDAC models and enhanced their sensitivity to gemcitabine. Together, our findings support a context-dependent role of the hyaluronan–CD44 axis in PDAC and suggest that differences in CD44 organization and hyaluronan responsiveness may contribute to functional heterogeneity among PDAC cellular states.

## Data Availability

All data generated or analyzed during this study are provided within the manuscript or Supplementary Information Files.

## Supplementary Materials

The following supporting information can be downloaded at: https://www.mdpi.com/article/10.3390/cancers18203410/s1, Additional experimental procedures and methodological details for qPCR, immunofluorescence and transcriptome sequencing. Primer sequences are listed in Table S1. Gene signatures were listed in Table S2; Figure S1 **CD44 expression is associated with EMT, poor prognosis, and basal-like PDAC.** (**A**). Kaplan-Meier survival curves showing the overall survival (OS) of patients with high vs. low *CD44* expression across PAAD, COAD, ESCA, LIHC, LUAD, LUSC, READ and STAD. (**B**). Representative TP63 IHC and CD44/E-cadherin (E-cad) IF co-localization in epithelial-like and mesenchymal-like patient tumor tissue. (**C**). Correlation between *CD44* and the EMT associated signature; r and *p* values shown. (**D**). Correlation between *CD44* and poor prognosis associated gene signature; r and P values shown. (**E**). GSEA showing the *Kras* signaling up and the *Kras* signaling down in *CD44* up-regulation and down-regulation tumors. (**F**). GSEA showing the *MYC* and *TGFβ* signaling association in *CD44* up-regulation and down-regulation tumors; Figure S2 **Histological Distribution, Prognostic Value, and Molecular Regulatory Network of CD44 in PDAC.** (**A**). Representative IHC images of CD44 in PDAC samples with different histological morphologies and stages. (**B**). Analysis of The Cancer Genome Atlas–PDAC/PAAD dataset for associations of *CD44*-high and *CD44*-low expression with overall survival (OS) at different grades (left: G1, G2, right: G3, G4). (**C**, **D**). *FN1* expression across PDAC molecular subtypes in the Bailey (GSE36924) and Moffitt (GSE71729) cohorts. (**E**, **F**). *LAM* protein family expression across PDAC molecular subtypes in the Bailey (GSE36924) and Moffitt (GSE71729) cohorts. **C**–**F**, Bailey (GSE36924) (Progenitor n = 21, Squamous n = 20) and Moffitt (GSE71729) (classical n = 132, basal-like n = 89) cohorts. unpaired Student *t* test, mean ± SD, ns, no significant, * *p* < 0.05, **** *p* < 0.0001. (**G**). Correlation between *CD44* and the cell polarity associated signature; r and *p* values shown. (**H**). Correlation between *CD44* and *CTNNB1*; r and *p* values shown. (**I**). Correlation between *CDH1* and *CTNNB1*; r and *p* values shown. (**J**). Immunoblot analysis of membrane and cytoplasmic fractions from BxPC-3 and MIA PaCa-2 cells; Figure S3 **CD44 regulates distinct signaling pathways in PDAC cells across molecular subtypes.** (**A**). KEGG Enrichment of CTR and *CD44*-KO groups in MIA PaCa-2 cells. (**B**). KEGG Enrichment of CTR and *CD44*-KO groups in CFPAC-1 cells. (**C**). GO and Pathway analysis of Pan02 control versus *CD44*-sh cells; Figure S4 **CD44 differentially regulates EMT-associated markers and migration in PDAC cells.** (**A**). Immunoblot analysis of ZEB1, SNAIL, TGFβR1 and COL1A1 expression in CTR and *CD44*-KO groups in MIA PaCa-2 and CFPAC-1 cells, GAPDH was used as an internal loading control. (**B**). Quantification of ZEB1, SNAIL, TGFβR and COL1A1 levels in *CD44*-KO cells relative to controls, Data represent mean ± SD for at least three independent experiments; unpaired Student *t* test, ns, no significant, ** *p* < 0.01, **** *p* < 0.0001. (**C**). Representative images and Quantification of migration assays of CTR and *CD44*-KO groups in CFPAC-1 cells. Data represent mean ± SD for at least three independent experiments; unpaired Student *t* test, ns, no significant. (**D**). Immunoblot analysis of CD44, ZEB1, Vimentin, and GAPDH in PANC-1 and its *CD44*-OE cells; Figure S5 **HA-related genes and stromal signals regulate CD44-associated signaling in PDAC cells.** (**A**). Kaplan–Meier survival analysis of PDAC patients based on the expression of HA synthesis-related genes (*HAS2* and *HAS3*) and HA degradation-related genes (*HYAL1* and *HYAL2*) from the Cancer Genome Atlas–PDAC/PAAD dataset. (**B**). Quantification analysis of time-course protein expression (0, 2, 5, 10, 15, and 30 min) following pretreatment with PP2 (10 µM), a SRC inhibitor, and stimulation with hyaluronic acid (HA: 50 mg/mL) in MIA-PACA-2, CFPAC-1, and MIA-*CD44*-KO cells. Target proteins included ZEB1, SNAIL, SRC, phosphorylated SRC (p-SRC), ERK, phosphorylated ERK (p-ERK), and the loading control GAPDH. Data represent mean ± SD for at least three independent experiments; unpaired Student *t* test, ns, no significant, * *p* < 0.05. (**C**). Quantification analysis of Hakai expression in different cells under the HA treatments. (**D**). Quantification analysis of CD44 protein expression of time-course protein expression (0, 2, 5, 10, 15, and 30 min) following stimulation with PaSCs cell supernatant in MIA PaCa-2 and CFPAC-1 cells. (**E**). Quantification analysis of CD44 protein expression of time-course protein expression (0, 1, 2 and 4 h) following stimulation with PaSCs cell supernatant in MIA PaCa-2 cells; Figure S6 **In vivo evaluation of treatment efficacy and systemic toxicity in orthotopic PDAC models.** (**A**). Schematic of orthotopic tumor xenograft models and drug administration in mice. (**B**). Representative H&E-stained images of livers from mice bearing orthotopic Pan02, MIA PaCa-2, or KPC tumors. (**C**, **E**, **G**). Mouse body weight in Pan02 (**C**), KPC (**E**), and MIA PaCa-2 (**G**) orthotopic tumors in each treatment group. (**D**, **F**, **H**). Liver, lung, and kidney weights in Pan02 (**D**), KPC (**F**), and MIA PaCa-2 (**H**) orthotopic tumors in each treatment group; Figure S7 **4-MU enhances gemcitabine sensitivity, and HA stimulation accelerates migration in basal-like PDAC cells.** (**A**). Cells were treated with single agents (4MU: 800, 400, 200, 100, 50 µM; GEM: 0.2, 0.1, 0.05, 0.025, 0.0125 µM) or a fixed-ratio combination (4-MU: GEM = 4000:1) for 72 h, and cell viability was assessed by MTT assay. CI and DRI values were calculated using CompuSyn software based on the Chou–Talalay method. Fa-CI plot, the horizontal dashed line indicates CI = 1 (additivity); CI < 1 indicates synergism. Fa-DRI plot, the horizontal dashed line indicates DRI = 1; DRI > 1 indicates a favorable dose reduction. Data are representative of three independent experiments. (**B**). Typical images and quantitative results of the scratch assay for MIA PaCa-2 cells in different groups (CTR = CTRL, HYAL = hyaluronic acid + hyaluronidase) (HA: 50 µg/mL) (hyaluronidase: 10µg /mL). Data are presented as mean ± SD from three independent experiments. unpaired Student *t* test, ** *p* < 0.01. (**C**). MTT assay showing the viability of MIA PaCa-2 cells treated with 4MU (200 µM) combined with gemcitabine (GEM, 0.01 µM) in the presence or absence of HA. Data are presented as mean ± SD from three independent experiments. unpaired Student *t* test, * *p* < 0.05.
