# Supplementary material for: Context-Dependent Spatial and Functional Roles of CD44 Across Pancreatic Ductal Adenocarcinoma States
Source: Cancers (Basel). 2026 Oct 21;18(20):3410. doi: 10.3390/cancers18203410 (PMC13702738; doi:10.3390/cancers18203410)
Supplement: Supplementary file 1 [file cancers-18-03410-s001.zip › cancers-4594753-supplementary.pdf]

Title: Context-dependent spatial and functional roles of CD44 across pancreatic ductal adenocarcinoma states

Authors: Bin Fu[#1], Yingying Tan[#1], Yaxin Chen[1], Yongren Su[2], Mengran Cao[1], Dashan Tan[1], Meng Zhang[1], Xuewei Chen[1], Rong Hu[1]

Affiliations:

1 State Key Laboratory of Natural Medicines, School of Basic Medicine and Clinical Pharmacy, China Pharmaceutical University, Nanjing, Jiangsu, P.R. China.

2 State Key Laboratory of Medicinal Chemical Biology and College of Pharmacy, Nankai University, Tianjin, China

Correspondence: ronghu@cpu.edu.cn.

# Contributed equally.

Supplementary Figures

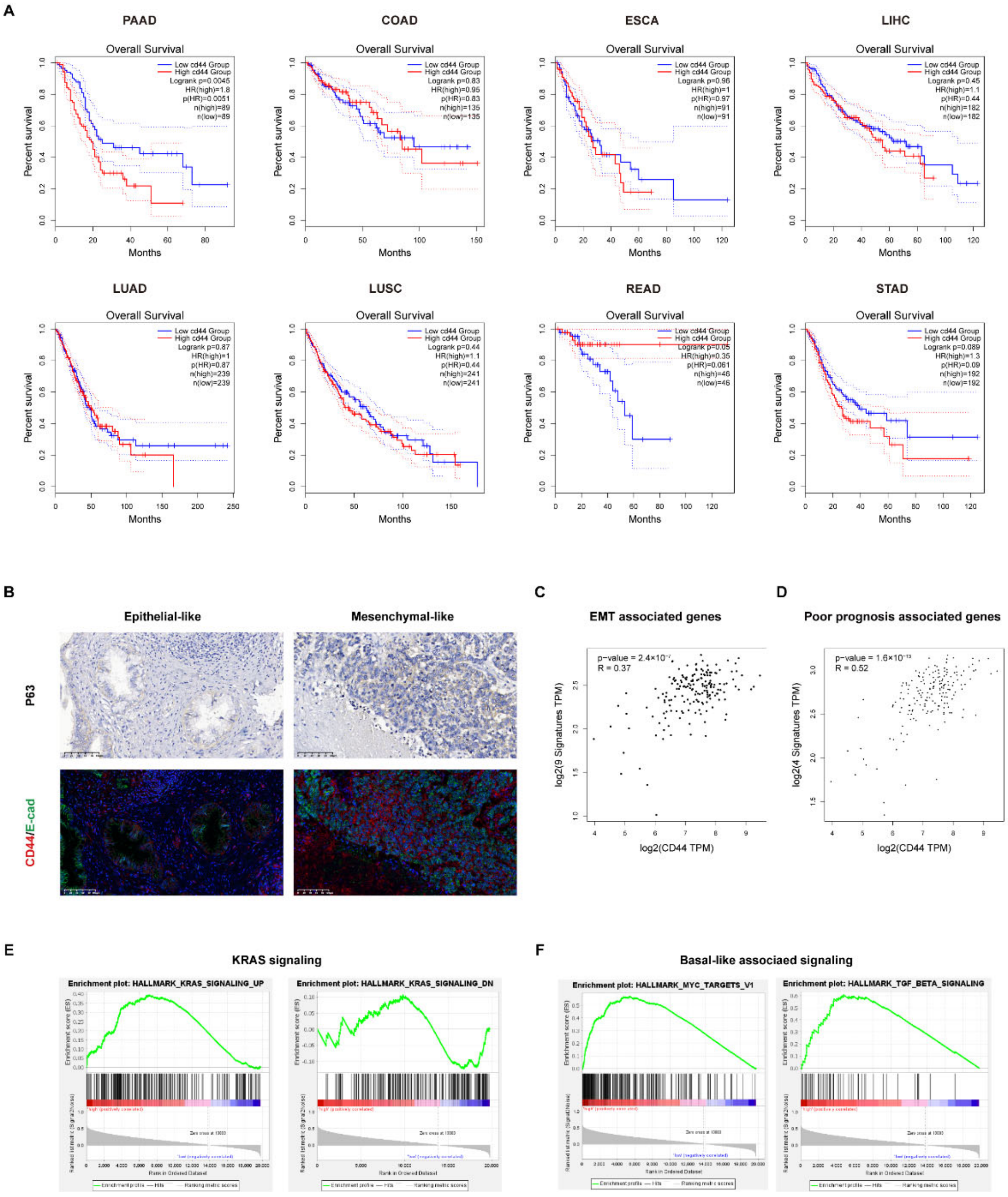

Supplementary Figure S1 CD44 expression is associated with EMT, poor prognosis, and basal-like PDAC.

(A). Kaplan-Meier survival curves showing the overall survival (OS) of patients with high vs. low *CD44* expression across PAAD, COAD, ESCA, LIHC, LUAD, LUSC,

READ and STAD. **(B)**. Representative TP63 IHC and CD44/E-cadherin (E-cad) IF co-localization in epithelial-like and mesenchymal-like patient tumor tissue. **(C)**. Correlation between *CD44* and the EMT associated signature; r and P values shown. **(D)**. Correlation between *CD44* and poor prognosis associated gene signature; r and P values shown. **(E)**. GSEA showing the *Kras* signaling up and the *Kras* signaling down in *CD44* up-regulation and down-regulation tumors. **(F)**. GSEA showing the *MYC* and *TGFβ* signaling association in *CD44* up-regulation and down-regulation tumors.

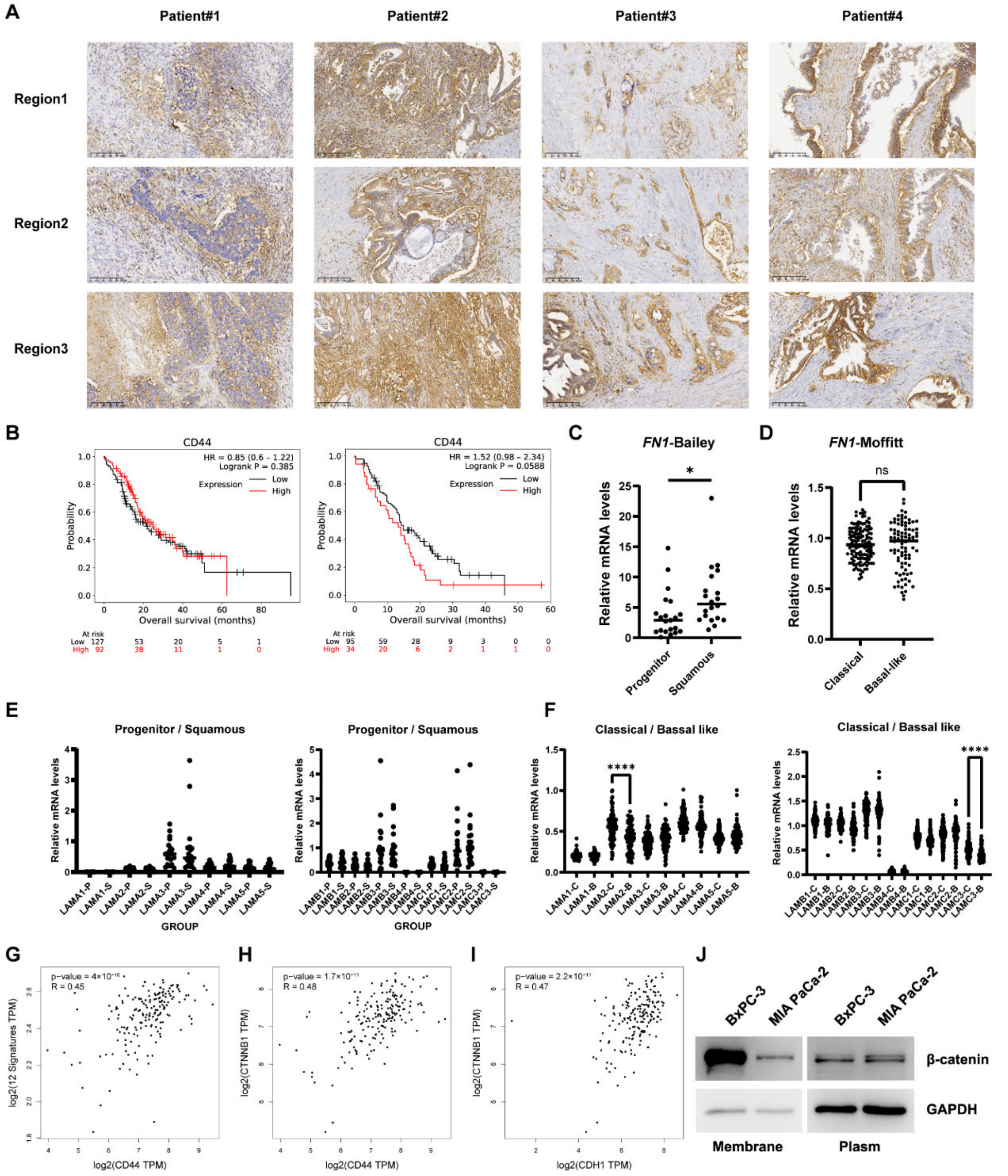

Supplementary Figure S2 Histological Distribution, Prognostic Value, and Molecular Regulatory Network of CD44 in PDAC

(A). Representative IHC images of CD44 in PDAC samples with different histological morphologies and stages. **(B)**. Analysis of The Cancer Genome Atlas–PDAC/PAAD dataset for associations of *CD44*-high and *CD44*-low expression with overall survival

(OS) at different grades (left: G1, G2, right: G3, G4). **(C, D)**. *FN1* expression across PDAC molecular subtypes in the Bailey (GSE36924) and Moffitt (GSE71729) cohorts. **(E, F)**. *LAM* protein family expression across PDAC molecular subtypes in the Bailey (GSE36924) and Moffitt (GSE71729) cohorts. **C – F**, Bailey (GSE36924) (Progenitor n = 21, Squamous n = 20) and Moffitt (GSE71729) (classical n = 132, basal-like n = 89) cohorts. unpaired Student t test, mean ± SD, ns, no significant, * P<0.05, **** P<0.0001. **(G)**. Correlation between *CD44* and the cell polarity associated signature; r and P values shown. **(H)**. Correlation between *CD44* and *CTNNB1*; r and P values shown. **(I)**. Correlation between *CDH1* and *CTNNB1*; r and P values shown. **(J)**. Immunoblot analysis of membrane and cytoplasmic fractions from BxPC-3 and MIA PaCa-2 cells.

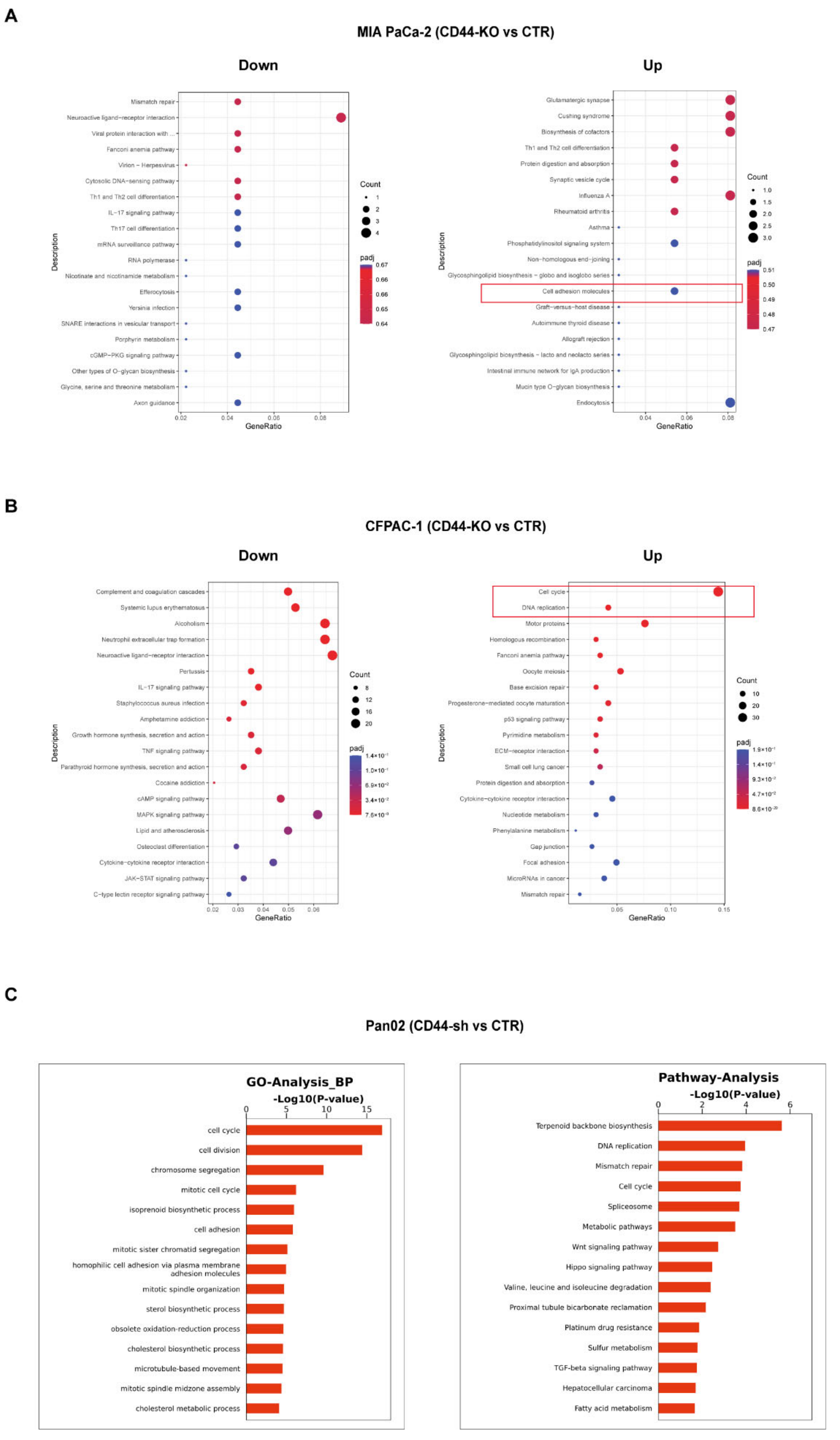
A
MIA PaCa-2 (CD44-KO vs CTR)
Down
Up
Description
GeneRatio
Count
padj
Cell adhesion molecules
B
CFPAC-1 (CD44-KO vs CTR)
Down
Up
Description
GeneRatio
Count
padj
Cell cycle
DNA replication
C
Pan02 (CD44-sh vs CTR)
GO-Analysis_BP
-Log10(P-value)
cell cycle
cell division
chromosome segregation
mitotic cell cycle
isoprenoid biosynthetic process
cell adhesion
mitotic sister chromatid segregation
homophilic cell adhesion via plasma membrane adhesion molecules
mitotic spindle organization
sterol biosynthetic process
obsolete oxidation-reduction process
cholesterol biosynthetic process
microtubule-based movement
mitotic spindle midzone assembly
cholesterol metabolic process
Pathway-Analysis
-Log10(P-value)
Terpenoid backbone biosynthesis
DNA replication
Mismatch repair
Cell cycle
Spliceosome
Metabolic pathways
Wnt signaling pathway
Hippo signaling pathway
Valine, leucine and isoleucine degradation
Proximal tubule bicarbonate reclamation
Platinum drug resistance
Sulfur metabolism
TGF-beta signaling pathway
Hepatocellular carcinoma
Fatty acid metabolism

Supplementary Figure S3 CD44 regulates distinct signaling pathways in PDAC cells across molecular subtypes

(A). KEGG Enrichment of CTR and *CD44*-KO groups in MIA PaCa-2 cells. **(B)**. KEGG Enrichment of CTR and *CD44*-KO groups in CFPAC-1 cells. **(C)**. GO and Pathway analysis of Pan02 control versus *CD44*-sh cells.

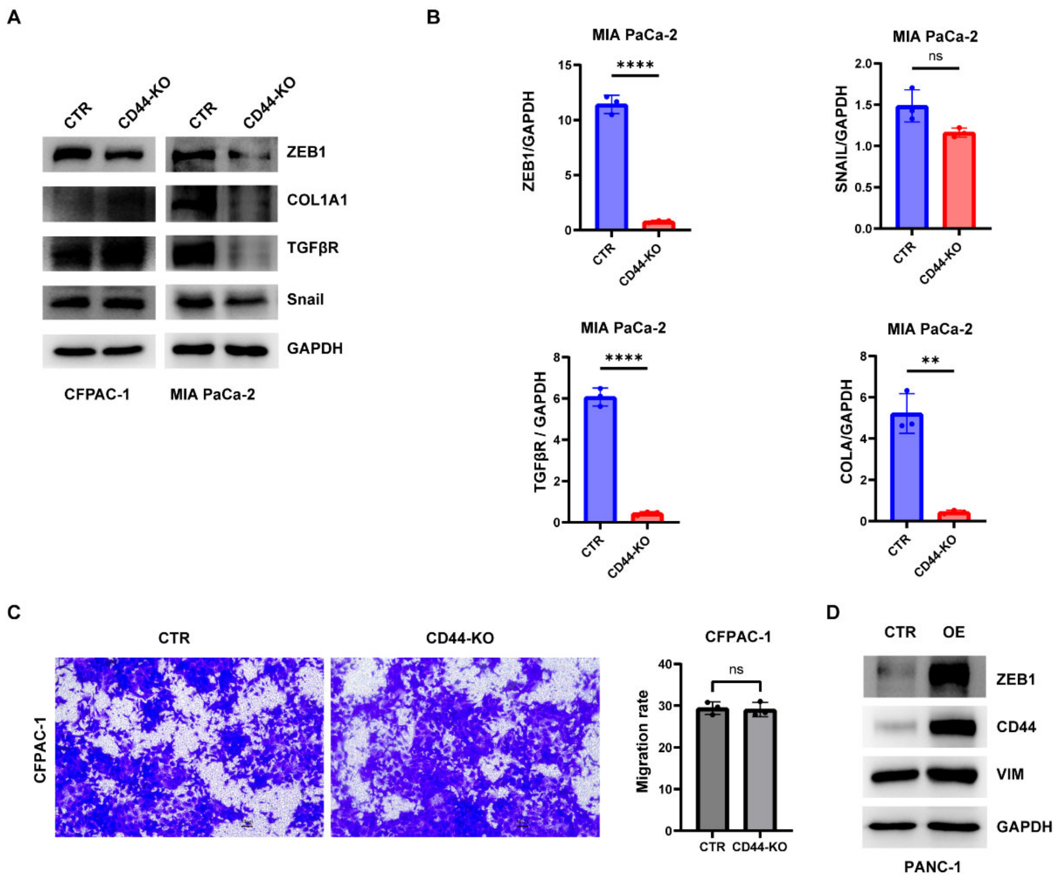

Supplementary Figure S4 CD44 differentially regulates EMT-associated markers and migration in PDAC cells

(A). Immunoblot analysis of ZEB1, SNAIL, TGFβR1 and COL1A1 expression in CTR

and *CD44*-KO groups in MIA PaCa-2 and CFPAC-1 cells, GAPDH was used as an internal loading control. **(B)**. Quantification of ZEB1, SNAIL, TGFβR and COL1A1 levels in *CD44*-KO cells relative to controls, Data represent mean ± SD for at least three independent experiments; unpaired Student t test, ns, no significant, ** $P<0.01$, **** $P<0.0001$. **(C)**. Representative images and Quantification of migration assays of CTR and *CD44*-KO groups in CFPAC-1 cells. Data represent mean ± SD for at least three independent experiments; unpaired Student t test, ns, no significant. **(D)**. Immunoblot analysis of CD44, ZEB1, Vimentin, and GAPDH in PANC-1 and its *CD44*-OE cells.

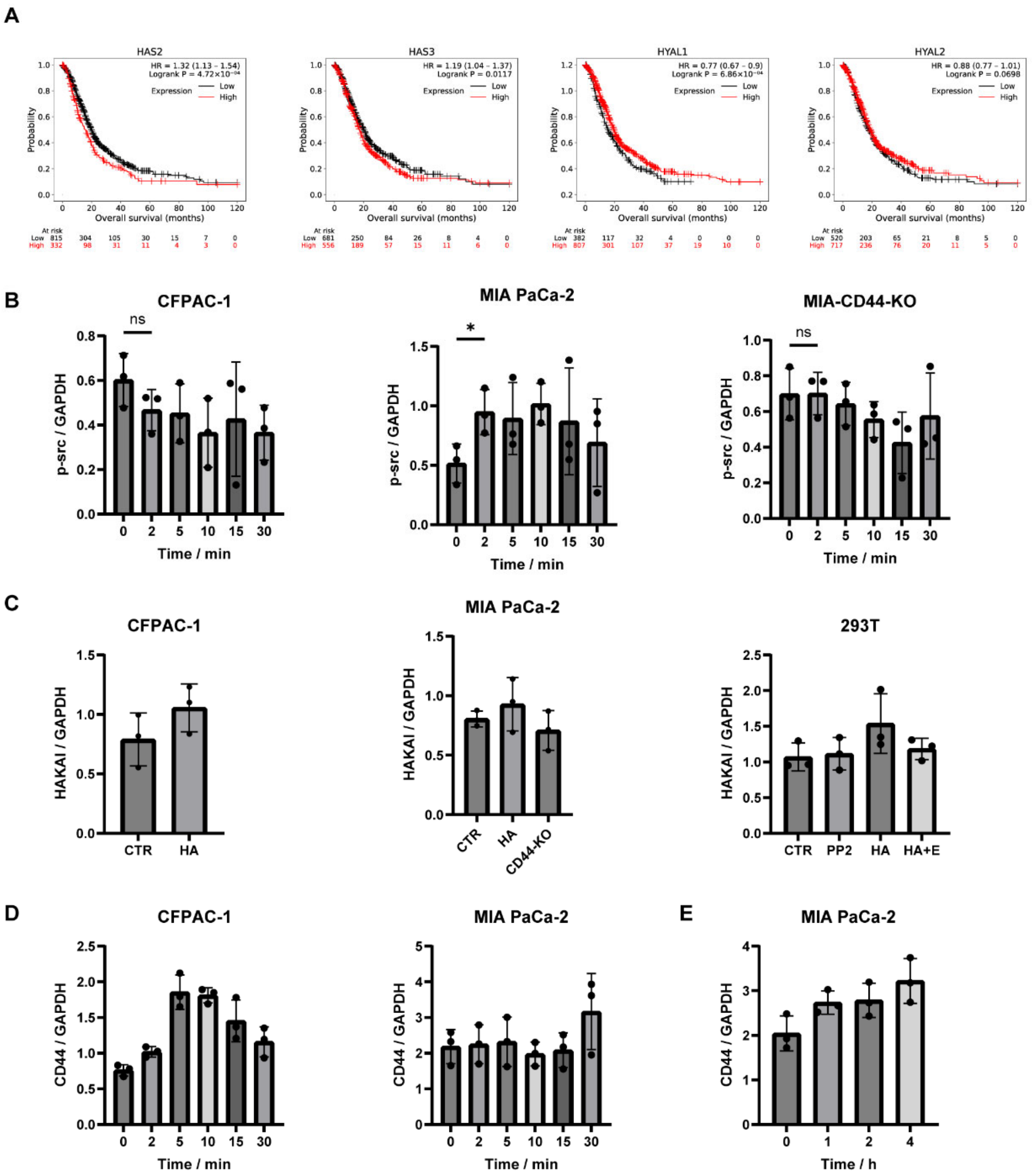

Supplementary Figure S5 HA-related genes and stromal signals regulate CD44-associated signaling in PDAC cells

(A). Kaplan–Meier survival analysis of PDAC patients based on the expression of HA synthesis-related genes (*HAS2* and *HAS3*) and HA degradation-related genes (*HYAL1* and *HYAL2*) from the Cancer Genome Atlas–PDAC/PAAD dataset. **(B)**. Quantification analysis of time-course protein expression (0, 2, 5, 10, 15, and 30 min) following

pretreatment with PP2 (10 μM), a SRC inhibitor, and stimulation with hyaluronic acid (HA: 50 mg/mL) in MIA-PACA-2, CFPAC-1, and MIA-*CD44*-KO cells. Target proteins included ZEB1, SNAIL, SRC, phosphorylated SRC (p-SRC), ERK, phosphorylated ERK (p-ERK), and the loading control GAPDH. Data represent mean ± SD for at least three independent experiments; unpaired Student t test, ns, no significant, * $P<0.05$. **(C)**. Quantification analysis of Hakai expression in different cells under the HA treatments. **(D)**. Quantification analysis of CD44 protein expression of time-course protein expression (0, 2, 5, 10, 15, and 30 min) following stimulation with PaSCs cell supernatant in MIA PaCa-2 and CFPAC-1 cells. **(E)**. Quantification analysis of CD44 protein expression of time-course protein expression (0, 1, 2 and 4 h) following stimulation with PaSCs cell supernatant in MIA PaCa-2 cells.

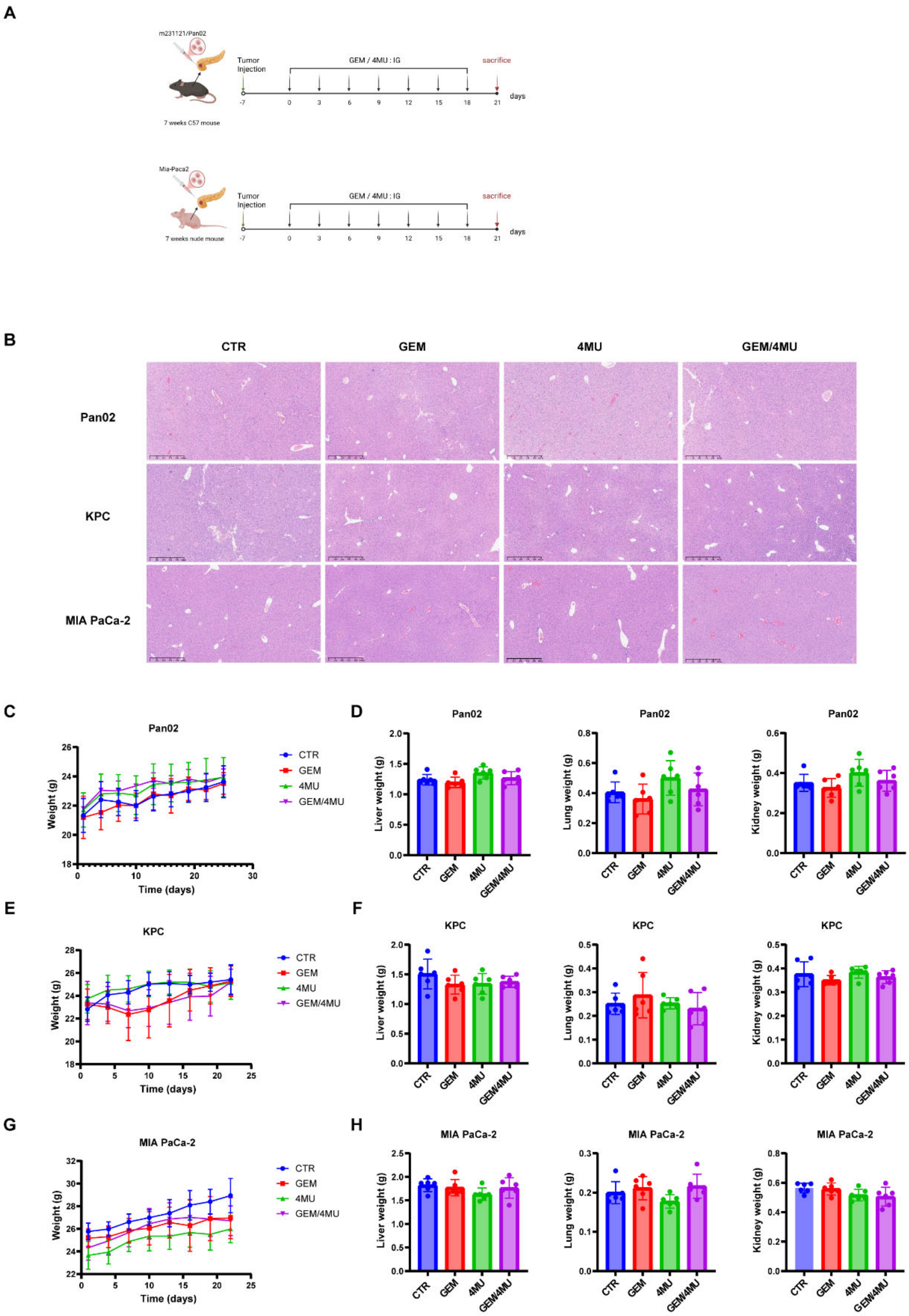

Supplementary Figure S6 In vivo evaluation of treatment efficacy and systemic toxicity in orthotopic PDAC models

(A). Schematic of orthotopic tumor xenograft models and drug administration in mice.

(B). Representative H&E-stained images of livers from mice bearing orthotopic Pan02, MIA PaCa-2, or KPC tumors. **(C, E, G)**. Mouse body weight in Pan02 **(C)**, KPC **(E)**, and MIA PaCa-2 **(G)** orthotopic tumors in each treatment group. **(D, F, H)**. Liver, lung, and kidney weights in Pan02 **(D)**, KPC **(F)**, and MIA PaCa-2 **(H)** orthotopic tumors in each treatment group.

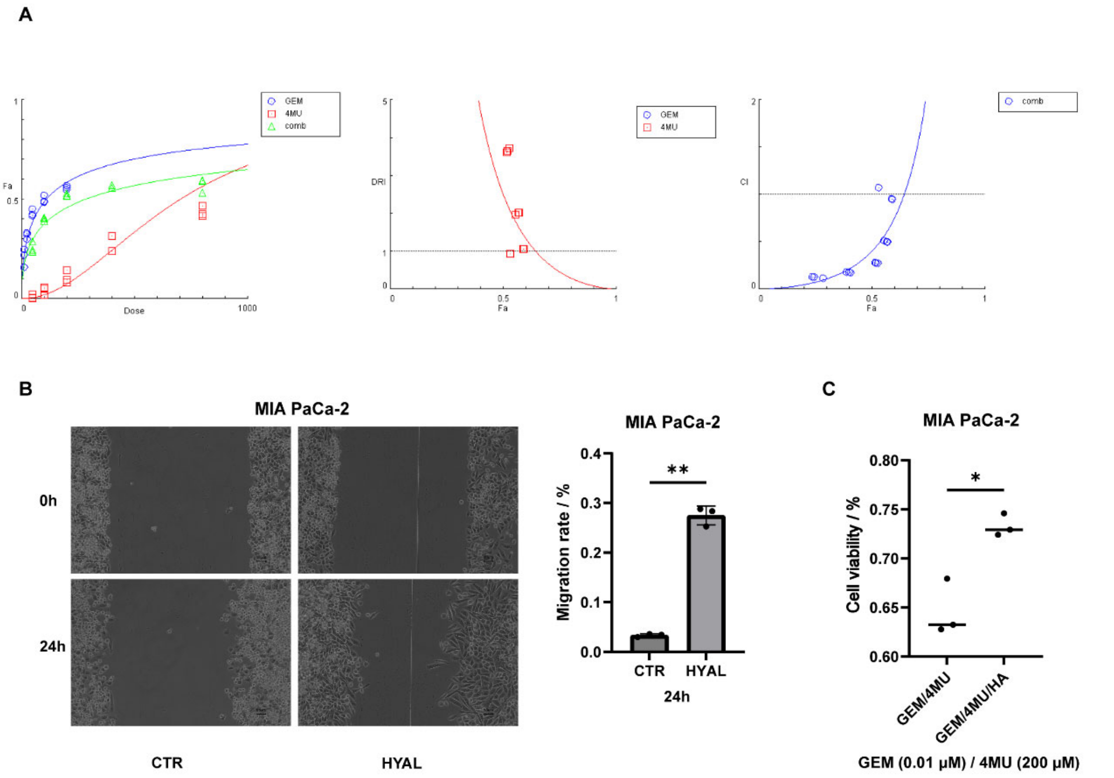

Supplementary Figure S7 4-MU enhances gemcitabine sensitivity, and HA stimulation accelerates migration in basal-like PDAC cells

A. Cells were treated with single agents (4MU: 800, 400, 200, 100, 50 μM; GEM: 0.2, 0.1, 0.05, 0.025, 0.0125 μM) or a fixed-ratio combination (4-MU: GEM = 4000:1) for 72 h, and cell viability was assessed by MTT assay. CI and DRI values were calculated

using CompuSyn software based on the Chou–Talalay method. Fa-CI plot, the horizontal dashed line indicates CI = 1 (additivity); CI < 1 indicates synergism. Fa-DRI plot, the horizontal dashed line indicates DRI = 1; DRI > 1 indicates a favorable dose reduction. Data are representative of three independent experiments. **B**. Typical images and quantitative results of the scratch assay for MIA PaCa-2 cells in different groups (CTR = CTRL, HYAL = hyaluronic acid + hyaluronidase) (HA: 50 μg/mL) (hyaluronidase: 10μg /mL). Data are presented as mean ± SD from three independent experiments. unpaired Student t test, ** P<0.01. **C**. MTT assay showing the viability of MIA PaCa-2 cells treated with 4MU (200 μM) combined with gemcitabine (GEM, 0.01 μM) in the presence or absence of HA. Data are presented as mean ± SD from three independent experiments. unpaired Student t test, * P<0.05.

Supplementary materials

qRT-PCR

Total RNA was extracted using TRIzol reagent (catalog no. 15596026, Life technologies) as described (). cDNA synthesis was performed by EasyScript First-Strand cDNA Synthesis Super Mix (catalog no. AE301, Transgen) with a reverse PCR instrument (TP600, TAKARA). Quantitative PCR was performed using Power SYBR Green PCR Master Mix (catalog no. Q421-02, Vazyme) on a CFX96 Real-Time PCR System (MA-6000, Suzhou Molarray Co., Ltd.). Primer sequences are listed in S1 table.

Table S1.

HAS1	Forward: CTGCGATACTGGGTAGCCTTCAATG
	Reverse: TAGAGGACCGCTGATGCAGGATAC
HAS2	Forward: GGGGCACATCAGGAAGGAAAACC
	Reverse: CACACCACCCAGGAGGATTGTAAAC
HAS3	Forward: CAAGGTGGTCATGGTGGTGGATG
	Reverse: CTCGCCTGCCTCATGGAAGTTG
HYAL1	Forward: CCTTAACCCTGCCAGTTTCTCCATC
	Reverse: TCGACATTTGAACTCCACAGCCATC
HYAL2	Forward: CCACAGCACCACCCATCTTCAC
	Reverse: ACATCAAAGGCATTCAGGTCCAGTG

Immunohistochemistry (IHC)

Paraffin-embedded tissue sections were deparaffinized in xylene, rehydrated through a graded ethanol series, and subjected to heat-induced antigen retrieval in EDTA buffer (pH 9.0). Endogenous peroxidase activity was blocked with 3% hydrogen peroxide, followed by serum blocking. Sections were incubated overnight at 4°C with primary antibodies against CD44 (CST, 37259S), p63 (Abways, CY5659), and β-catenin (Abways, CY3523) at a dilution of 1:300. After washing, sections were incubated with HRP-conjugated secondary antibodies and developed using 3,3′-diaminobenzidine (DAB). Slides were counterstained with hematoxylin, dehydrated, and mounted. Images were captured using a light microscope, and staining was evaluated

independently by investigators blinded to the experimental groups.

Immunofluorescence (IF)

For clinical tissue samples, Paraffin-embedded tissue sections were deparaffinized, rehydrated, and subjected to heat-induced antigen retrieval in EDTA buffer (pH 9.0). After serum blocking, sections were incubated overnight at 4°C with primary antibodies against CD44 (AiFang Biological, AF20017) and E-cadherin (AiFang Biological, AF01810). Fluorescent signals were detected using HRP-conjugated secondary antibodies followed by tyramide signal amplification (TSA)-based staining according to the manufacturer's instructions. For sequential staining, an additional antigen retrieval step was performed between antibody incubations. Nuclei were counterstained with DAPI, and slides were mounted using antifade mounting medium.

For MIA-PACA2 and CFPAC1 cells, cells were seeded onto glass coverslips and cultured to 60–80% confluence. Cells were fixed with 4% paraformaldehyde for 15 min at room temperature, washed with PBS, and permeabilized with 0.1% Triton X-100 for 5 min when required. After blocking with 5% bovine serum albumin (BSA) for 1 h, cells were incubated overnight at 4°C with primary antibodies against CD44 (Immunoway, YM4707) and E-cadherin (HUABIO, HA723564). Following PBS washes, cells were incubated with species-specific Alexa Fluor-conjugated secondary antibodies for 1 h at room temperature in the dark. Nuclei were counterstained with DAPI, and coverslips were mounted using antifade mounting medium.

Images were acquired using a confocal or fluorescence microscope.

RNA Sequencing and Data Analysis

For PAN02 and PAN02-CD44-sh cells

RNA Extraction and Library Preparation

Total RNA was extracted from samples using TRIzol reagent (Invitrogen, USA) and quality assessed on an Agilent 2200 Bioanalyzer. Samples with an RNA integrity number (RIN) > 7.0 were used for cDNA library construction. Libraries were prepared using the VAHTS Universal V6 RNA-seq Library Prep Kit for Illumina (Vazyme, Nanjing, China) according to the manufacturer's instructions. Briefly, poly(A) mRNA was purified from 1 μg total RNA using oligo(dT) magnetic beads and fragmented into 200–600 bp fragments with divalent cations at 85 °C for 6 min. First- and second-strand cDNA were synthesized using dUTP for second-strand synthesis to enable strand-specificity. cDNA fragments were end-repaired, A-tailed, and ligated to indexed adapters. After purification, the second strand was removed by uracil DNA glycosylase treatment, and first-strand cDNA was enriched by PCR to generate sequencing libraries. Library quality was assessed using the Agilent 2200 Bioanalyzer, and paired-end 150 bp sequencing was performed on the DNBSEQ-T7 platform.

Read Mapping and Quantification

Raw reads were processed to remove adapter sequences and low-quality reads. Clean reads were aligned to the mouse reference genome (mm10) using STAR. Gene-level counts were obtained with HTSeq, and expression levels were calculated using the RPKM method.

Differential Gene Expression Analysis

Differentially expressed genes (DEGs) were identified using DESeq2. Genes with fold change > 2 or < 0.5, P-value < 0.05, and false discovery rate (FDR) < 0.05 were considered significant.

Gene Ontology and Pathway Analysis

Gene ontology (GO) enrichment analysis was performed to identify the biological functions of DEGs. GO annotations were retrieved from NCBI, UniProt, and the Gene Ontology Consortium. Significant GO terms were identified using Fisher’s exact test (P < 0.05). Pathway enrichment analysis was performed based on the KEGG database, with significance determined using Fisher’s exact test (P < 0.05).

For MIA-PACA2, CFPAC1 and their CD44-KO cells

Sample collection and preparation

RNA quantification and qualification

①RNA degradation and contamination was monitored on 1% agarose gels.

② RNA purity was checked using the NanoPhotometer® spectrophotometer (IMPLEN, CA, USA).

③RNA integrity was assessed using the RNA Nano 6000 Assay Kit of the Bioanalyzer 2100 system (Agilent Technologies, CA, USA).

Library preparation for Transcriptome sequencing

A total amount of 1 μg RNA per sample was used as input material for the RNA sample

preparations. Sequencing libraries were generated using NEBNext® UltraTM RNA Library Prep Kit for Illumina® (NEB, USA) following manufacturer's recommendations and index codes were added to attribute sequences to each sample. Briefly, mRNA was purified from total RNA using poly-T oligo-attached magnetic beads. Fragmentation was carried out using divalent cations under elevated temperature in NEBNext First Strand Synthesis Reaction Buffer(5X). First strand cDNA was synthesized using random hexamer primer and M-MuLV Reverse Transcriptase (RNase H-). Second strand cDNA synthesis was subsequently performed using DNA Polymerase I and RNase H. Remaining overhangs were converted into blunt ends via exonuclease/polymerase activities. After adenylation of 3' ends of DNA fragments, NEBNext Adaptor with hairpin loop structure were ligated to prepare for hybridization. In order to select cDNA fragments of preferentially 250~300 bp in length, the library fragments were purified with AMPure XP system (Beckman Coulter, Beverly, USA). Then 3 μl USER Enzyme (NEB, USA) was used with size-selected, adaptor-ligated cDNA at 37°C for 15 m in followed by 5 min at 95 °Cbefore PCR. Then PCR was performed with Phusion High -Fidelity DNA polymerase, Universal PCR primers and Index (X) Primer. At last, PCR products were purified (AMPure XP system) and library quality was assessed on the Agilent Bioanalyzer 2100 system.

Clustering and sequencing (GenechemExperimental Department)

The clustering of the index-coded samples was performed on a cBot Cluster Generation System using TruSeq PE Cluster Kit v3-cBot-HS (Illumia) according to the manufacturer's instructions. After cluster generation, the library preparations were

sequenced on an Illumina Novaseq platform and 150 bp paired-end reads were generated.

Data Analysis

Quality control

Raw data (raw reads) of fastq format were firstly processed through in-house perl scripts. In this step, clean data (clean reads) were obtained by removing reads containing adapter, reads containing ploy-N and low quality reads from raw data. At the same time, Q20, Q30 and GC content the clean data were calculated. All the downstream analyses were based on the clean data with high quality.

Reads mapping to the reference genome

Reference genome and gene model annotation files were downloaded from genome website directly. Index of the reference genome was built using Hisat2 v2.0.5 and paired-end clean reads were aligned to the reference genome using Hisat2 v2.0.5. We selected Hisat2 as the mapping tool for that Hisat2 can generate a database of splice junctions based on the gene model annotation file and thus a better mapping result than other non-splice mapping tools.

Quantification of gene expression level

featureCounts v1.5.0-p3 was used to count the reads numbers mapped to each gene. And then FPKM of each gene was calculated based on the length of the gene and reads count mapped to this gene. FPKM, expected number of Fragments Per Kilobase of

transcript sequence per Millions base pairs sequenced, considers the effect of sequencing depth and gene length for the reads count at the same time, and is currently the most commonly used method for estimating gene expression levels.

Differential expression analysis

(For DESeq2 with biological replicates) Differential expression analysis of two conditions/groups (two biological replicates per condition) was performed using the DESeq2 R package (1.16.1). DESeq2 provide statistical routines for determining differential expression in digital gene expression data using a model based on the negative binomial distribution. The resulting P-values were adjusted using the Benjamini and Hochberg's approach for controlling the false discovery rate . Genes with an adjusted P-value <0.05 found by DESeq2 were assigned as differentially expressed. (For edgeR without biological replicates) Prior to differential gene expression analysis, for each sequenced library, the read counts were adjusted by edgeR program package through one scaling normalized factor. Differential expression analysis of two conditions was performed using the edgeR R package (3.18.1). The P values were adjusted using the Benjamini & Hochberg method. Corrected P-value of 0.05 and absolute foldchange of 2 were set as the threshold for significantly differential expression.

GO and KEGG enrichment analysis of differentially expressed genes

Gene Ontology (GO) enrichment analysis of differentially expressed genes was implemented by the clusterProfiler R package, in which gene length bias wascorrected.

GO terms with corrected Pvalue less than 0.05 were considered significantly enriched by differential expressed genes.

KEGG is a database resource for understanding high-level functions and utilities of the biological system, such as the cell, the organism and the ecosystem, from molecular-level information, especially large-scale molecular datasets generated by genome sequencing and other high-through put experimental technologies (http://www.genome.jp/kegg/). We used clusterProfiler R package to test the statistical enrichment of differential expression genes in KEGG pathways.

SNP analysis

GATK2 (v3.7) software was used to perform SNP calling. Raw vcf files were filtered with GATK standard filter method and other parameters (cluster:3; WindowSize:35; QD < 2.0 o; FS > 30.0; DP < 10 and SnpEff software was used to annotation for the Variablesite.

AS analysis

Alternative Splicing is an important mechanism for regulate the expression of genes and the variable of protein. rMATS(3.2.5) software was used to analysis the AS event.

PPI analysis of differentially expressed genes

PPI analysis of differentially expressed genes was based on the STRING database, which known and predicted Protein-Protein Interactions.

Fusion Analysis

Fusion gene refers to the chimeric gene formed by the fusion of all or part of the sequences of two genes, which is generally caused by chromosome translocation, deletion and other reasons. We used Starfusion software (1.2.0) to detect genes that are fused. Star-fusion is a software package uses fusion output results of STAR alignment to detect fusion transcripts, including SATR alignment, SATRfusion. predict, SATR-fusion.filter was used to correct the predicted results of Star-fusion to ensure the accuracy of the results.

Weighted correlation network analysis

WGCNA (Weighted correlation network analysis) is a systematic biological method used to describe the gene association modes among different samples. it can be used to identify gene sets that are highly synergistic changed, and identify candidate biomarkers or therapeutic targets based on the coherence of gene sets and the correlation between gene sets and phenotypes. The R package WGCNA is a set of functions used to calculate various weighted association analysis, which can be used for network construction, gene screening, gene cluster identification, topological feature calculation, data simulation and visualization. WGCNA is suitable for multi-sample data. Generally, more than 15 samples are required. One input file is sample information, that is, a matrix describing the traits of the sample: the traits used for association analysis must be numeric; If it is a regional or categorical variable, it needs to be converted to a 0-1 matrix. The other is gene expression data. For transcriptome sequencing, FPKM can be used as gene expression data.

Gene Set Enrichment Analysis

Gene Set Enrichment Analysis (GSEA) is a computational approach to determine if a pre-defined Gene Set can show a significant consistent difference between two biological states. The genes were ranked according to the degree of differential expression in the two samples, and then the predefined Gene Set were tested to see if they were enriched at the top or bottom of the list. Gene set enrichment analysis can include subtle expression changes. We use the local version of the GSEA analysis tool http://www.broadinstitute.org/gsea/index.jsp, GO, KEGG, Reactome, DO, DisGeNET data set were used for GSEA independently.

Dataset analysis

Correlation analysis was generated from Gepia2 (http://gepia2.cancer-pku.cn/#index) Public Library of Bioinformatics. Gene signatures were listed in S2.

Table S2.

Gene set	GENES
Classical associated signature	*GATA6, HNF1A, HNF4A, FOXA2, MUC1, MUC13*
Basal-like associated signature	*KRT5, KRT6A, KRT14, KRT17, TP63, LAMC2, ITGA6, S100A2*
EMT associated signature	*CDH2, VIM, FN1, SNAI1, SNAI2, ZEB1, ZEB2, TWIST1, TWIST2*

Poor prognosis associated signature	*AMIGO2, KLK6, PMEPA1, TGM2*
Cell polarity associated signature	*PARD3, PARD6A, PARD6B, PRKCI, PRKCZ, TJP1 (ZO1), TJP2, CLDN1, CLDN3, OCLN, CTNNB1, CTNND1*

Supplementary Cell viability and combination index analysis

Cells were seeded in 96-well plates at a density of 3×10^3 cells per well and allowed to adhere overnight. The following day, cells were treated with single-agent gemcitabine (0.0125, 0.025, 0.05, 0.1 and 0.2 μM), single-agent 4-methylumbelliferone (50, 100, 200, 400, and 800 μM), or a fixed-ratio combination of both agents for 72 h. The combination was prepared at a constant 4-MU:gemcitabine ratio of 4000:1. Cell viability was assessed using the MTT assay according to the manufacturer's instructions. Relative cell viability was calculated as:

$$\text{Cell viability (\%)} = \frac{A_{\text{treated}}}{A_{\text{control}}} \times 100$$

All experiments were performed in triplicate. The fraction affected (Fa) was calculated as Fa = 1 − (cell viability / 100). Dose-response curves for each single agent were fitted using the median-effect equation, and the combination index (CI) was calculated using CompuSyn software (ComboSyn, Inc., Paramus, NJ, USA) based on the Chou–Talalay method. CI values < 1, = 1, and > 1 indicate synergism, additivity, and antagonism, respectively. Dose-reduction index (DRI) values were also derived from CompuSyn to quantify the fold reduction in the dose of each agent required to achieve the same effect in combination versus single-agent treatment.

Supplementary Wound Healing Assay

MIA PaCa-2 cells were seeded at equal densities in 12-well plates and cultured to 90–100% confluence. To exclude the contribution of cell proliferation to wound closure, mitomycin C (10 μg/ml; Psaitong, M70002) was added to each well and incubated for 2 h before wounding. A uniform linear wound was then created using a sterile 100-μL pipette tip. Cells were washed with PBS to remove detached cells and cultured in serum-free medium.

Cells were assigned to two treatment groups: (1) control (CTR), receiving vehicle only; and (2) HYAL group, treated with HA (50 μg/mL) and hyaluronidase (10 μg/mL). Images of the same wound area were captured at 0 and 24 h using an inverted microscope.

Each experiment was performed with three biological replicates, and three fields were analyzed per replicate. Wound closure was quantified using ImageJ software, and the relative migration rate was calculated based on the initial wound area at 0 h. Data are presented as mean ± SD from three independent experiments. unpaired Student t test, ** $P<0.01$.

Supplementary HA rescue experiment

Cells were seeded in 96-well plates at 3×10^3 cells per well and allowed to adhere overnight. Cells were then divided into two groups: a control group and an HA-supplemented group. Both groups were treated with gemcitabine (0.01 μM) or 4-methylumbelliferone (200 μM) for 72 h, with HA added to the HA-supplemented group. Cell viability was assessed using the MTT assay according to the manufacturer's instructions. Data are presented as mean ± SD from three independent experiments. unpaired Student t test, * $P<0.05$
